# DPSCs regulate epithelial-T cell interactions in oral submucous fibrosis

**DOI:** 10.1186/s13287-024-03720-5

**Published:** 2024-04-23

**Authors:** S. Y. Wang, S. J. Zhang, H. F. Meng, H. Q. Xu, Z. X. Guo, J. F. Yan, J. L. Gao, L. N. Niu, S. L. Wang, K. Jiao

**Affiliations:** 1 Department of Stomatology, Tangdu Hospital & State Key Laboratory of Oral and Maxillofacial Reconstruction and Regeneration & School of Stomatology, The Fourth Military Medical University, 169 West Changle Road, Xincheng District, 710032 Xi’an, Shaanxi P. R. China; 2 State Key Laboratory of Oral and Maxillofacial Reconstruction and Regeneration & National Clinical Research Center for Oral Diseases & Shaanxi Key Laboratory of Stomatology, School of Stomatology, The Fourth Military Medical University, 169 West Changle Road, Xincheng District, 710032 Xi’an, Shaanxi P. R. China; 3 Beijing SH Bio-tech Co., 100071 Beijing, P.R. China; 4 The College of Life Science, Northwest University, 710032 Xi’an, Shaanxi P.R. China; 5 Beijing Laboratory of Oral Health, Capital Medical University, 10 Xitoutiao, Fengtai District, 100069 Beijing, P.R. China; 6 Laboratory of Homeostatic Medicine, School of Medicine, Southern University of Science and Technology, No. 1088 Xueyuan Avenue, Nanshan District, 518055 Shenzhen, P.R. China

**Keywords:** Cellular microenvironment, Dental pulp, Stem cells, Epithelial cells, Inflammation, Fibrosis, Single-cell gene expression analysis

## Abstract

**Background:**

Oral submucous fibrosis (OSF) is a precancerous lesion characterized by fibrous tissue deposition, the incidence of which correlates positively with the frequency of betel nut chewing. Prolonged betel nut chewing can damage the integrity of the oral mucosal epithelium, leading to chronic inflammation and local immunological derangement. However, currently, the underlying cellular events driving fibrogenesis and dysfunction are incompletely understood, such that OSF has few treatment options with limited therapeutic effectiveness. Dental pulp stem cells (DPSCs) have been recognized for their anti-inflammatory and anti-fibrosis capabilities, making them promising candidates to treat a range of immune, inflammatory, and fibrotic diseases. However, the application of DPSCs in OSF is inconclusive. Therefore, this study aimed to explore the pathogenic mechanism of OSF and, based on this, to explore new treatment options.

**Methods:**

A human cell atlas of oral mucosal tissues was compiled using single-cell RNA sequencing to delve into the underlying mechanisms. Epithelial cells were reclustered to observe the heterogeneity of OSF epithelial cells and their communication with immune cells. The results were validated *in vitro*, in clinicopathological sections, and in animal models. *In vivo*, the therapeutic effect and mechanism of DPSCs were characterized by histological staining, immunohistochemical staining, scanning electron microscopy, and atomic force microscopy.

**Results:**

A unique epithelial cell population, Epi1.2, with proinflammatory and profibrotic functions, was predominantly found in OSF. Epi1.2 cells also induced the fibrotic process in fibroblasts by interacting with T cells through receptor-ligand crosstalk between macrophage migration inhibitory factor (MIF)-CD74 and C-X-C motif chemokine receptor 4 (CXCR4). Furthermore, we developed OSF animal models and simulated the clinical local injection process in the rat buccal mucosa using DPSCs to assess their therapeutic impact and mechanism. In the OSF rat model, DPSCs demonstrated superior therapeutic effects compared with the positive control (glucocorticoids), including reducing collagen deposition and promoting blood vessel regeneration. DPSCs mediated immune homeostasis primarily by regulating the numbers of KRT19^ +^ MIF ^+^ epithelial cells and via epithelial-stromal crosstalk.

**Conclusions:**

Given the current ambiguity surrounding the cause of OSF and the limited treatment options available, our study reveals that epithelial cells and their crosstalk with T cells play an important role in the mechanism of OSF and suggests the therapeutic promise of DPSCs.

**Supplementary Information:**

The online version contains supplementary material available at 10.1186/s13287-024-03720-5.

## Background

Fibrotic diseases are characterized by excessive growth of fibrous connective tissue and a decrease in parenchymal cells within organs and tissues. This process is often triggered by inflammation and, if left uncontrolled, can cause structural damage and organ malfunction, ultimately leading to organ sclerosis. Oral submucous fibrosis (OSF) is a fibrotic disease affecting the oral mucosa, potentially progressing to oral squamous cell carcinoma (OSCC) [1]. It manifests as pale, hardened oral mucosa and restricted mouth opening. Oral submucous fibrosis carries a risk of malignant transformation, ranging from 7.6 to 13% [2]. There are limited treatment options for OSF, mainly comprising surgery and local submucosal glucocorticoid (GC) injections [3]. However, to better manage this chronic disease, a deeper understanding of the mechanisms regulating OSF and the development of novel therapeutic approaches are required.

Betel nut chewing significantly contributes to OSF; however, its development involves multiple, as yet incompletely understood, factors [3, 4]. The abrasive fibers in betel nuts can damage oral mucosal epithelial cells, leading to chronic inflammation and alteration of the local immune environment, eventually culminating in fibrosis. Clinical evidence also confirms the correlation between inflammation and fibrosis. Pathological sectioning of OSF tissues revealed extensive inflammatory cell infiltration, and clinical grading indicated inflammation as a prefibrotic stage [5]. Damage to the oral mucosal epithelium is the initial factor of inflammation. Epithelial and immune cells have roles in fibrotic disease pathogenesis [6]; however, the interaction between these cells in OSF progression remains underexplored, creating a significant knowledge gap.

Mesenchymal stem cells (MSCs) have immunoregulatory properties and are effective to treat inflammatory and fibrotic diseases, including Type 2 Diabetes and periodontitis [7–9]. Dental pulp stem cells (DPSCs), are easily obtained, have low immunogenicity, do not pose ethical concerns, and thus have been used to treat liver fibrosis, lung fibrosis, and kidney fibrosis [10–12]. Considering the similar pathological features between these conditions and OSF, we hypothesized that DPSCs might be employed to treat OSF, while avoiding the side effects associated with GCs and surgery (e.g., insomnia, epilepsy, depression, and scars) [13–16].

Herein, we identified the role of epithelial cell populations and the interactions in the oral mucosal tissues of patients with OSF using single-cell RNA sequencing (scRNA-seq). The results were verified using *in vivo* and *in vitro* experiments and the impact of DPSCs on OSF rats was evaluated. By linking these mechanisms with a potential therapy, we aimed to provide a useful clinical strategy to manage OSF.

## Methods

### Collection of tissue samples

The study conformed to the STROBE guidelines. Participants must be over 18 and in good health. Patients were excluded who had blood diseases, a history of Hepatitis B or C, HIV, active malignancy, recent treatment with certain medications, or autoimmune disorders within 1 year. Oral health evaluation was conducted alongside systemic screening to ensure that only individuals without soft tissue lesions and no indication of oral or dental infection were included in the study. The inclusion criteria for OSF were clinical stage III to IV (< 20 mm mouth opening and/ or accompanied by leukoplakia or OSCC). The tissue samples used for analysis in this study were obtained from 19 participants who were diagnosed with OSF in the last three years. In addition, samples of standard human oral mucosa were collected from six patients who were either receiving treatment for mucosal hyperplasia or undergoing tissue resection as part of orthognathic surgery procedures. One fresh tissue sample from each group was sent for scRNA-seq analysis, while the remaining samples were made into sections, stained, and observed. T cells were sourced from four additional blood samples. It is important to note that all tissue samples were histopathologically diagnosed by the Pathology Department of the Air Force Medical University (AFMU) in Xi’an, Shaanxi Province, China. Detailed, written, informed consent was obtained from all volunteers in accordance with protocols approved by the ethical committee of the AFMU (IRB-REV-2022049; IRB-REV-2022058). Supplementary Table 1 provides information on the clinical parameters of the healthy volunteers and patients with OSF.

### Preparation of samples for scRNA-seq

Upon collection, tissue samples were transported in sterilized culture dishes containing 10 mL of 1× Dulbecco’s Phosphate-Buffered Saline (DPBS; Thermo Fisher Scientific, Cat. no. 14190144) under chilled conditions to wash away residual tissue storage solution. The tissues were subsequently minced under similar cold conditions. A combination of 0.25% Trypsin (Thermo Fisher Scientific, Cat. no. 25200-072) and 10 µg/mL of DNase I (Sigma, Cat. no. 11284932001) were employed, each dissolved in phosphate-buffered saline (PBS) containing 5% Fetal Bovine Serum (FBS; Thermo Fisher Scientific, Cat. no. A3161001C), to facilitate tissue digestion. Human tissues underwent dissociation at a controlled temperature of 37 ℃, with an oscillation speed of 50 rpm maintained over approximately 40 min. To optimize cell yield and viability, the dissociated cells were intermittently harvested every 20 min. The resultant cell suspensions were filtered through a 40 μm nylon cell strainer. Furthermore, any red blood cells present were eliminated using 1× Red Blood Cell Lysis Solution (Thermo Fisher Scientific, Cat. no. 00-4333-57). Dissociated cells were rinsed with 1× DPBS incorporating 2% FBS. To assess cell viability, cells were stained with 0.4% Trypan blue (Thermo Fisher Scientific, Cat. no.15250061) and enumerated using a Countess® II Automated Cell Counter (Thermo Fisher Scientific, Waltham, MA, USA).

### Preparation of the 10× library and the sequencing procedure

Each bead was characterized by a unique molecular identifier (UMI) and cell barcodes were loaded close to the saturation point. This ensured that each individual cell was suitably paired with a bead in a Gel Beads-in-Emulsion setup. Following exposure to a cell lysis buffer, polyadenylated RNA molecules were hybridized with the beads. The beads were subsequently collected into a single tube for subsequent reverse transcription. During cDNA synthesis, each cDNA molecule received a tag on its 5' end, which corresponds to the 3' end of a messenger RNA transcript. This tagging mechanism incorporated a UMI and cell label to trace the cell of origin for each molecule. The 10× beads were then subjected to second-strand cDNA synthesis, adapter ligation, and universal amplification. Sequencing libraries were assembled using the products of randomly interrupted whole-transcriptome amplification. This approach facilitated the enrichment of the 3' end of the transcripts, which were linked with the cell barcode and UMI. Following these stages, all remaining procedures, including library construction, were performed according to the standard manufacturer’s protocol (10× Genomics, Pleasanton, CA, USA). Sequencing libraries were quantified using a High Sensitivity DNA Chip (Agilent, Santa Clara, CA, USA) on a Bioanalyzer 2100 instrument with the Qubit High Sensitivity DNA Assay (Thermo Fisher Scientific). Finally, the prepared libraries were sequenced on a NovaSeq6000 (Illumina, San Diego, CA, USA) platform, utilizing 2 × 150 chemistry.

### Processing and quality control of the scRNA-seq data

Reads were handled using the Cell Ranger 2.1.0 pipeline (10× Genomics), applying default and suggested parameters. FASTQ files generated from Illumina sequencing output were analyzed using the STAR algorithm [17]. Gene-barcode matrices were generated for each distinct sample through UMI counting and the filtering of non-cell associated barcodes. Consequently, a gene-barcode matrix was constructed, encompassing barcoded cells and corresponding gene expression counts. This output was then incorporated into the Seurat (v2.3.0) R toolkit to enable quality control and further analysis of the scRNA-seq data [18]. All functions were executed using default parameters, except where explicitly stated otherwise. Cells exhibiting fewer than 200 or more than 6000 detected genes (each gene necessitating at least one UMI aligned in a minimum of three cells) were excluded. The expression of mitochondrial genes was computed utilizing the PercentageFeatureSet function of the Seurat package [18]. Cells with mitochondrial gene expression exceeding 10% were excluded to eliminate low-activity cells. Data normalization (via the NormalizeData function in the Seurat package) was performed to extract a subset of variable genes, which were then identified while controlling for the strong correlation between variability and average expression. Data from diverse samples were subsequently integrated, following the identification of ‘anchors’ between datasets utilizing FindIntegrationAnchors and IntegrateData in the Seurat package [18]. Principal Component Analysis (PCA) was performed, and the data were reduced to the top 30 PCA components following scaling. The resulting clusters were visualized on a two dimensional (2D) map constructed using t-distributed Stochastic Neighbor Embedding (t-SNE).

### Identification of cell types and subtypes via nonlinear dimensional reduction (t-SNE)

Cells were clustered using graph-based clustering of the PCA-reduced data, following the computation of a shared nearest neighbor graph using the Louvain Method [18]. For sub-clustering, the same procedure (scaling, dimensional reduction, and clustering) was applied to a specific dataset, typically restricted to one cell type. For each cluster, the Wilcoxon Rank-Sum Test was employed to identify significantly differentially expressed genes when compared with the remaining clusters. Cell types were then ascertained using SingleR and known marker genes [19].

### Pseudotime analysis

Pseudotime analysis was conducted utilizing the Monocle2.14.0 software [20]. This analysis incorporated all subsets of epithelial cells and facilitated cell order sorting using the subgroup markers procured from previous analyses. This allowed for a holistic evaluation of the expression change relationship between subsets. The DDRTree algorithm was employed to reduce the dimension of known differentiation-related genes, genes exhibiting significant coefficients of variation, and the differential gene data of subsets. The differentiation time for each cell was calculated, a starting point was randomly selected, and the cells were sorted accordingly. Differentiation-related genes were then calculated based on the time of differentiation.

### Cell–cell interaction analysis

To visualize and analyze intercellular communications derived from scRNA-seq data, a cellchat analysis was carried out [21]. A new cellchat object was created from the existing Seurat object, with cell types added to the cellchat object as cell metadata. Cellchat identified differentially overexpressed ligands and receptors for each cell group and linked each interaction with a probability value, thereby quantifying communications between two cell groups mediated by these signaling molecules. Significant interactions were recognized based on a statistical test that randomly permuted the group labels of cells and recalculated the interaction probability. A ligand or receptor was classified as ‘expressed’ in a particular cell cluster if it was present in more than 25% of cells.

### Immunohistochemistry and immunofluorescence staining of tissue

For immunohistochemistry, tissue sections were deparaffinized, rehydrated, and antigen retrieval was performed using a citrate buffer. Sections were blocked with a protein-blocking solution and incubated with primary antibodies overnight at 4 °C and then with horseradish peroxidase-conjugated secondary antibodies for 1 h at room temperature. The 3,3′-Diaminobenzidine (DAB) substrate was added until the desired stain intensity developed. The antibodies used in this study recognized keratin 19 (KRT19) (1:1000, 60187-1-lg, Proteintech), alpha smooth muscle actin (α-SMA) (1:300, GTX100034, GeneTex), CD31 (also known as platelet and endothelial cell adhesion molecule 1 (PECAM1)) (1:200, GB113151, Servicebio), and CD3 (1:1000, 17617-1-AP, Proteintech). For immunofluorescence staining, tissue sections were deparaffinized, rehydrated, blocked, and then incubated with primary antibodies overnight at 4 °C. Fluorescently tagged secondary antibodies were then applied. To visualize cell nuclei, sections were counterstained using 4′,6-diamidino-2-phenylindole (DAPI) (Invitrogen). Digital images were captured utilizing a fluorescence microscope (Nikon A1R; Nikon Corporation, Tokyo, Japan). The integrated fluorescence intensity across these images was quantified using Image Pro Plus 6.0 software (Media Cybernetics, Rockville, MA, USA). The antibodies employed in this study included those recognizing KRT19 (1:500, 60187-1-lg, Proteintech), macrophage migration inhibitory factor (MIF) (1:300, 20415-1-AP, Proteintech), CD3 (1:300, 17617-1-AP, Proteintech), CD74 (1:500, GTX110477, Proteintech), and C-X-C motif chemokine receptor 4 (1:100, 60042-1-Ig, Proteintech).

### Isolation and activation of T cells

In this study, peripheral blood mononuclear cells (PBMCs) were retrieved from the venous blood of healthy volunteers using Ficoll density gradient centrifugation. To isolate T cells, a specific number of PBMCs were resuspended in 100 µL of separation buffer and subjected to sorting using the MojoSort Human CD3 T Cell Isolation Kit (BioLegend; Cat. no. 480022). To sort the CD3 ^+^ T cells, 10 µL of CD3 negative antibody was added to each 1 × 10^7^ cells. Once sorted, the cells were transferred to X-VIVO15 medium (serum-free hematopoietic cell medium) supplemented with 5% human serum for T cell culture. To activate the CD3 ^+^ T cells, CD3/CD28 magnetic beads (Dynabeads Human T-activator CD3/CD28, Life Technologies, Cat. no. 11131D) were employed. The activated CD3 ^+^ T cells were then utilized in experiments conducted a week later.

### Cell culture

Various types of cells were used in the experimental procedures, including human oral epithelial keratinocytes (HOK-16B, zl-040906, ZLZT, Wuhan, China) and human gingival fibroblasts (HGFs, CP-H205, Procell). T cells and human DPSCs were also employed. DPSCs were sourced from Beijing SH Biotechnology (Beijing, China; http://www.bjshbio.com/) and their isolation and culture followed established methods [22, 23]. Cells (HGFs and DPSCs) from passages 4–6 were used to ensure consistency and reliability. Additionally, all cell lines were confirmed to be free of mycoplasma infection. To create the Epi1.2 cell model, HOK cells were cultured in an environment with 60 µg/mL arecoline for 24 h. Furthermore, all cell cultures were maintained in a 5% CO_2_ atmosphere at 37 °C to support optimal growth and functionality.

### Cell migration assay

HOK cells were propagated to confluence in a 6-well plate, after which a sterile 10 µL pipette tip was used to introduce a scratch down the center of each well. Detached cells were cleared via washing, and the cells were subsequently incubated in fresh medium. Images were captured at the 0, 24, and 48 h time points using an inverted microscope, which permitted an evaluation of cell migration into the scratched area. The formula used was [Wound healing percentage = (initial wound area−wound area at a certain point in time) / initial wound area] [24].

### Cell proliferation assay

Human oral epithelial cells were sown at a density of 2,000 cells per well in a 96-well plate and incubated for 24 h and 48 h. Following this, the culture medium was replaced with a medium reflecting different conditions (Control: Dulbecco’s modified Eagle’s medium (DMEM); Arecoline: medium containing varying concentrations of arecoline (20, 40, 60, and 80 µg/mL), respectively). The cell proliferation rate was evaluated at the 0, 24, and 48 h, using a Cell Counting Kit-8 assay (Elabscience; Cat. no. E-CK-A362). The absorbance at 450 nm was recorded using a microplate reader (BIO-TEK, Winooski, VT, USA).

### Immunofluorescence assay

Cells were fixed with 4% paraformaldehyde for 30 min. Following this, the cells were incubated with serum for 30 min to block non-specific binding sites. The primary antibody was added and incubated overnight after removal of the serum. The secondary antibody was then introduced and allowed to interact with the cells for 1 h, shielded from light. DAPI was subsequently added to stain the cell nuclei. The specimens were then observed using confocal scanning laser microscopy (Nikon A1R; Nikon Corporation). Integrated fluorescence intensity was quantified using ImageJ software (National Institute of Health, Bethesda, MD, USA). The antibodies employed in this study recognized KRT19 (1:300, 60187-1-lg, Proteintech), MIF (1:300, 20415-1-AP, Proteintech), CD3 (1:100, 17617-1-AP, Proteintech), CD74 (1:500, GTX110477, Proteintech), C-X-C motif chemokine receptor 4 (1:100, 60042-1-Ig, Proteintech) and α-SMA (1:500, GTX100034, GeneTex).

### Flow cytometry assay

Human T cells, T cells co-cultured with Epi1.2 cells, and DPSCs were digested into single cell suspensions. T cell activation was assessed by flow cytometry staining for cell-surface expression of CD69 and CD25. Intracellular flow cytometry staining of granzyme B was conducted by fixing and permeabilizing cells with Cytofix/Cytoperm Fixation/Permeabilization Solution Kit (BD Biosciences; Cat. no. 554714). To determine the number of proliferating T cells, T cells and T cells co-cultured with Epi1.2 cells were incubated for 30 min at 37 °C in the dark with CytoTell Red 650 (AAT Bioquest, Cat. no. 22255). The following antibodies were used for flow cytometry staining: phycoerythrin (PE)-anti-human CD25 (Biolegend, 302606), Allophycocyanin (APC)-anti-human CD69 (Biolegend, 310910), fluorescein isothiocyanate (FITC) anti-human/mouse Granzyme B (Biolegend, 396404). For DPSC phenotype identification, anti-human monoclonal antibodies targeting specific surface markers were used, including PE-anti-human CD73 (BioLegend, 344,003), PE-anti-human CD90 (BioLegend, 328109), PE-anti-human CD105 (BioLegend, 323205), PE-anti-human CD11b (BioLegend, 982606), PE-anti-human CD34 (BioLegend, 343505), PE-anti-human CD45 (BioLegend, 304007), FITC-anti-human CD19 (BioLegend, 302205), and FITC-anti-human CD3 (BioLegend, 300,306). Following incubation, the cells were resuspended and kept in the dark. Flow cytometry (Coulter-XL; Beckman Coulter, Indianapolis, IN, USA) was used to detect the cell surface markers, and the data analysis was performed using FlowJo 10.0 software (Flow Jo LLC, Ashland, OR, USA) or EXPO32 ADC Analysis software (Beckman Coulter).

### Transwell assay

These experiments were performed in 12-well Transwell plates with a 0.4 μm pore membranes (NEST; Cat. no. 724101). Human CD3 ^+^ T cells (1 × 10^6^ ) were seeded to the upper compartment of the chamber, while Epi1.2 cells (2 × 10^6^) were seeded to the lower compartment. Cells were cultured for 24 h and the number of T cells in the lower chamber was recorded for analysis.

### *In vitro*suppression assays

HOK cells were seeded in 6-well plates at 2 × 10^6^ /mL. After cell adherence, 60 µg/mL arecoline medium was added to three wells and cultured for 24 h to induce Epi1.2 cells. The MIF inhibitor ISO-1 (1 µM) (HY-16692, Med Chem Express) was dissolved in dimethyl sulfoxide according to the manufacturer’s instructions and added into the cell culture medium. After incubation for 24 h, T cells (1 × 10^6^) were added to the 6-well plates. Immunofluorescence and flow cytometry were used to detect Epi1.2 cells and T cells after 24 h of co-culture.

### Enzyme-linked immunosorbent assay (ELISA)

In this study, HOK cells, Epi1.2 cells, T cells, and Epi1.2 + T cells were cultivated in 48-well plates for 24 h. Subsequently, the supernatant from each group was collected and used to detect various cytokines, including interleukin (IL)-6 (Servicebio, GEH0001), IL-1β (Servicebio, GEH0002), IL-17 (MultiSciences; EK117/2–96), transforming growth factor beta (TGF-β) (MultiSciences, EK981-96), tumor necrosis factor alpha (TNF-α) (Servicebio, GEH0004), and interferon gamma (IFN-γ) (Servicebio, GEH0006). The quantification of these cytokines was performed using ELISA kits according to the manufacturer’s guidelines. The absorbance at 450 nm was measured using the microplate reader.

### Quantitative real-time reverse transcription polymerase chain reaction (qRT-PCR)

The supernatants of HOK cells, Epi1.2 cells, T cells, Epi1.2 + T cells and ISO-1 + Epi1.2 + T cells were collected, subjected to high-speed centrifugation, and the supernatants were co-cultured with HGFs. Total RNA was extracted from HGFs using the TRIzol reagent (Invitrogen). Subsequently, cDNA synthesis was carried out using a PrimeScript RT reagent kit (Takara Bio, Inc., Shiga Japan). Quantitative real-time PCR was performed using the cDNA as the template in a 7500 Real-Time PCR System (Applied Biosystems, Foster City, CA, USA). PCR amplification was conducted using genespecific primers and a high-fidelity DNA polymerase, with *GAPDH* (encoding glyceraldehyde-3-phosphate dehydrogenase) serving as the internal control. The 2^−ΔΔCt^ method was used to calculate the fold changes in gene expression relative to the control group [25]. For the *ACTA2* (actin alpha 2, smooth muscle (α-SMA)) gene, the forward primer sequence was 5-'TGGAAAAGATCTGGCACCAC-3', and the reverse primer sequence was 5'TCCGTTAGCAAGGTCGGATG-3'.

### Multipotential differentiation

The differentiation of DPSCs into multiple cell types was achieved by using osteogenic (ScienCell; Cat. no.7531) and adipogenic test kits (ScienCell; Cat. no.7541) in accordance with the manufacturer’s instructions. After the induction process, the cells were fixed using 4% paraformaldehyde and subsequently stained with alizarin red S (Sigma-Aldrich) for osteogenesis or Oil Red O (Sigma-Aldrich) for adipogenesis.

### Establishment of animal models, interventions, and measurements

The study conformed to the updated ARRIVE 2.0 Guidelines. Eight-week-old male Sprague-Dawley rats were obtained from the Laboratory Animal Research Center at AFMU, following approval from the Animal Experiment Administration Committee of the University (No. 2021-010). The rats were anesthetized with a gas anesthesia machine (R540, RWD, Shenzhen, China). The rats were placed in an induction box, and anesthesia was induced with a concentration of 3-4% isoflurane. The depth of anesthesia was assessed by shaking the induction box, and if the rat was unable to turn back on its own, it was indicated that the rat was under anesthesia. The rats were removed and placed in a prone position, and anesthesia was maintained by nasal inhalation of 1-2.5% isoflurane at a gas flow rate of 0.5–0.7 L/min. At the end of the experiment, isoflurane was turned off and the rats were allowed to breathe in pure oxygen for 5–10 min to wake up. The animal model was established by local application of arecoline. Arecoline (SA9640, Solarbio, 10 mg/ mL) was first dissolved in PBS. To create an animal model, a brush with bristles made of Dupont silk (9 mm in length and 0.02 mm in diameter) was used. The brush, which had a head diameter of 6 mm, was dipped into a solution of 15 mg/mL arecoline and applied to both sides of the buccal mucosa. A pressure of 6 N was exerted on the brush in a direction perpendicular to the mucosa, while each side of the buccal mucosa was subjected to 40 strokes. Following application, the animals were fasted for two h. After 12 weeks of daily treatment, a stable white lesion developed in the buccal mucosa of the rats, indicating the presence of OSF. The OSF animal models were then randomly divided into four intervention groups: OSF, OSF + PBS, OSF + GC/PBS, and OSF + DPSC/PBS (*n* = 6 per group). The negative control group received PBS treatment, while the positive control group received glucocorticoids (GC/PBS, 20 mg/mL). DPSCs were resuspended in 100 µL of PBS at a concentration of 1 × 10^6^ cells/mL. Each intervention group received weekly intra-cheek injections of 50 µL of the assigned treatment for either 4 or 8 weeks. Throughout the intervention period, weekly measurements were taken for body weight, mouth opening, and the area of the buccal lesion under isoflurane anesthesia. Measurements were carried out using procedures described previously [16]. Specifically, sulfate paper was laid over the lesion site in the oral mucosa of the rats, and the size and shape of the lesion were clearly presented on the paper. After that, the sulfate paper was removed and placed on grid paper to measure and record the specific dimensions of the lesion. All animals were kept in an isolated room with constant temperature and humidity. At the end of the intervention period, after rats were anesthetized with isoflurane inhalation, all animals were euthanized by cervical dislocation and oral mucosal tissues were collected for subsequent analysis. The time endpoint for animal modeling was 12 weeks, and the time endpoints for injection intervention were 4 and 8 weeks. All experimental procedures were carried out in accordance with the “Animal Research: Reporting of In Vivo Experiments” guidelines for preclinical animal studies.

### Histological staining

Oral mucosal tissue samples were fixed in 4% paraformaldehyde for 24 h, dehydrated through a graded ethanol series, cleared with xylene, and embedded in paraffin. Section (5 μm thickness) were obtained using a microtome. Histological staining was carried out using Hematoxylin and Eosin (H&E), Masson’s Trichrome (Masson) and Sirius Red staining. Image analysis of the stained sections was conducted using ImageJ software.

### Scanning electron microscopy (SEM)

The samples for SEM were initially fixed with 2.5% glutaraldehyde. Following fixation, the samples underwent a dehydration process using a series of graded ethanol concentrations. Once dehydrated, the samples were air-dried and subsequently sputtercoated with a thin layer of gold to their enhance surface conductivity, allowing for better imaging. The surface topography of the samples was visualized using a field-emission scanning electron microscope (FE-SEM, S-4800; Hitachi, Tokyo, Japan) operating at an acceleration voltage of 5 kV. To analyze the SEM images, ImageJ software was employed. This software facilitated the calculation of porosity based on the obtained SEM images.

### Atomic force microscopy (AFM)

Paraffin sections were analyzed using an atomic force microscope (Keysight 5500; Keysight Technologies, Santa Rosa, CA, USA) operating in tapping mode. Images were captured using a silicon probe (PPP-NCLR-20; Nanosensors, Neuchatel, Switzerland) with a 42-N/m force constant and a 161-kHz resonance frequency. To assess mechanical properties, a probe (SD-Sphere-CONT-m-10; Nanosensors) with a 0.2-N/m force constant and a 13-kHz resonance frequency was used, achieving a 300-nm indentation depth at each section position. Six different locations within each tissue sample were measured and then averaged for analysis.

### Preparation of conditioned media

DPSCs were seeded at 3 × 10^6^/ml and when achieving 80-90% confluence, the medium was changed to a serum-free DMEM (Gibco, Rockville, MD, USA). After incubation for 48 h, the medium was collected, centrifuged at 2500 rpm for 3 min, and then filtered through 0.22-µm pore filters (Millex®-GP; Merck Millipore Ltd., Billerica, MA, USA).

### Statistical analysis

The data were analyzed using GraphPad Prism 9 software (GraphPad Inc., La Jolla, CA, USA). The values are presented as the mean ± standard deviation (SD). All experiments were conducted independently at least three times. To assess the statistical significance between two groups, Student’s *t-*test was employed. For comparisons involving multiple groups, either one-way or two-way analysis of variance (ANOVA) was performed, followed by Tukey’s test. The significance levels were defined as *P < 0.05, **P < 0.01, ***P < 0.001, and ****P < 0.0001.

## Results

### A subpopulation of epithelial cells, Epi1.2, was increased in OSF

**Fig. 1:**
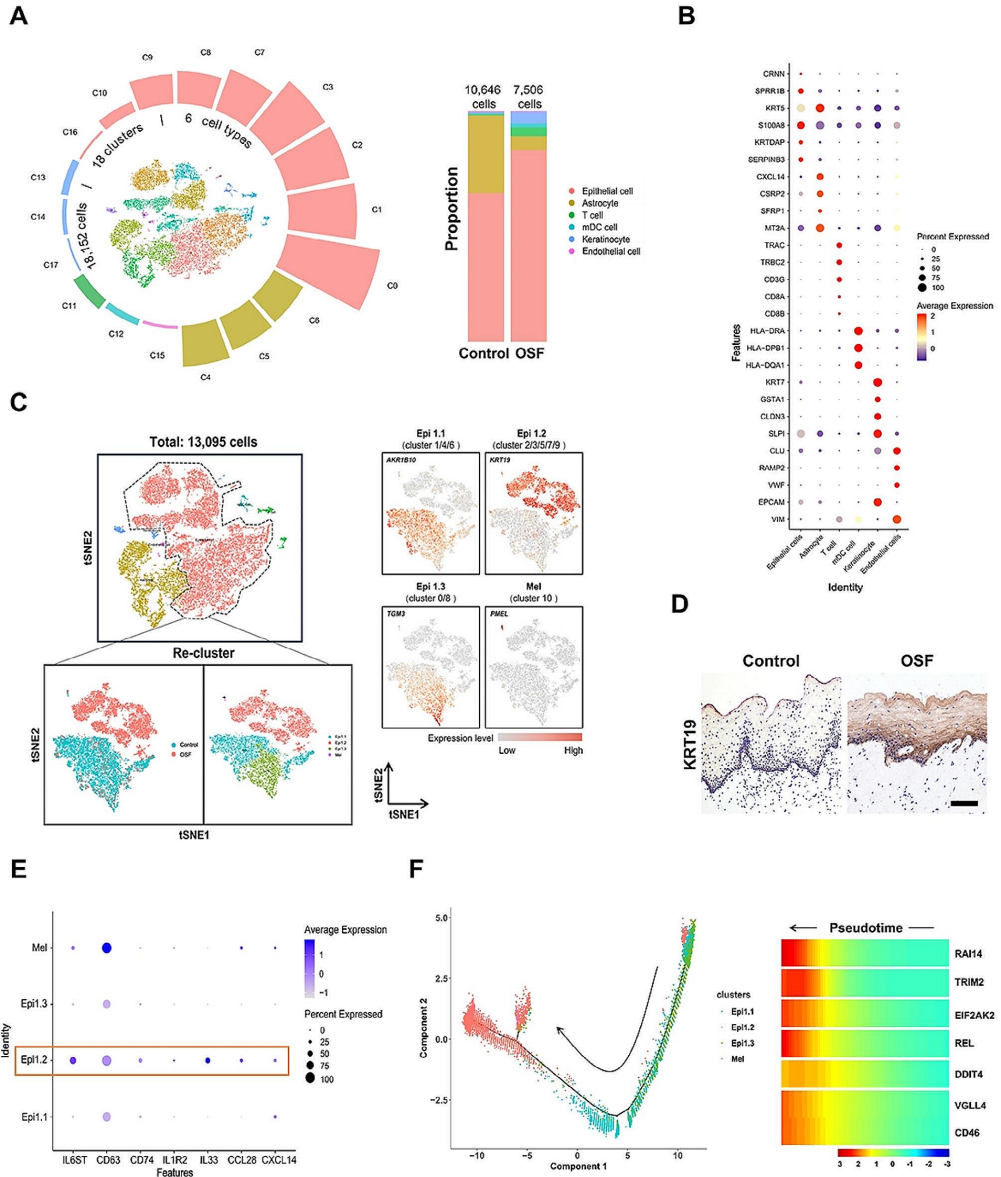
Single-cell profiling of OSF and epithelial cell subpopulation characterization. **(A)** Total cell subcluster analysis was performed using t-distributed stochastic neighborhood embedding (t-SNE). T-SNE presentation (center) shows 18,152 cells profiled from two individual human oral mucosa from healthy (10,646 cells) and OSF (7,506 cells) conditions. The surrounding circular layouts indicate the cell proportion of each cluster and six major cell types (different colors) (left) and a bar graph of relative cell proportions by tissue type (right). **(B)** Bubble plots depicting cell type marker gene expression. **(C)** Epithelial cell subpopulations with the distribution between the samples (left) and associated marker genes (right). **(D)** Levels of Epi1.2 cells (marked by KRT19) in control and OSF clinical samples. Scale bar = 100 μm. **(E)** Bubble plots illustrate the average gene expression of inflammatory factors and chemokines across epithelial cell subpopulations. The presence of Epi1.2 cells is highlighted by an orange box. **(F)** Pseudotime trajectory plots for epithelial cells by subpopulations. The black arrow points to the potential Epi1.2 cells activation trajectory. The heatmap displays gene expression changes along the Epi1.2 cells differentiation trajectory using Monocle. Red signifies higher expression; blue is lower.

To construct a comprehensive cellular and molecular map of OSF, a detailed analysis of the transcriptome derived from a multitude of individual cells was undertaken. These cells were obtained from the oral mucosal tissues of patients who had undergone routine health checks or OSF-specific surgical procedures (Appendix Fig. 1). To ensure the reliability of the data, we implemented rigorous quality control measures. This involved excluding low-quality cells, which were identified by their high expression of mitochondrial gene signatures, as well as removing any potential doublets. In total, 10,646 cells from the control group and 7,506 cells from the OSF group were included in the analysis (Appendix Fig. 2A). We identified 18 clusters that could be categorized into six distinct cell types (Fig. 1A). The cell clusters were meticulously annotated, drawing upon previously reported marker gene expression data [26, 27]. The marker genes for each cell type are shown in Fig. 1B. Specifically, we identified oral mucous epithelial cells (*CRNN*, *SPRR1B*, *S100A8, KRTDAP*, *SERPINB3*), astrocytes (*KRT5*, *CXCL4*, *CSRP2*, *MT2A*, *SFRP1*), T cells (*TRAC*, *TRBC2*, *CD3G*, *CD8A, CD8B)*, myeloid dendritic cells (mDCs) *(HLA-DPB1*, *HLA-DRA*, *HLA-DQA1*), keratinocytes (*KRT7*, *GSTA1*, *CLDN3*, *SLPI*, *EPCAM*), and endothelial cells (*CLU*, *RAMP2*, *VWF*, *VIM*) (Fig. 1B). Compared with the control group, the proportion of epithelial cells (Control *vs.* OSF: 64.5% *vs.* 83.0%), keratinocytes (Control *vs.* OSF: 0% *vs.* 4.9%) and T cells (Control *vs.* OSF: 0.4% *vs.* 3.9%) presented a potentially increased trend (without statistical analysis) because of the limited sample source (Fig. 1A). This observation provided significant insights into the cellular dynamics associated with OSF and laid the groundwork for further exploration and understanding of this condition.

**Fig. 2:**
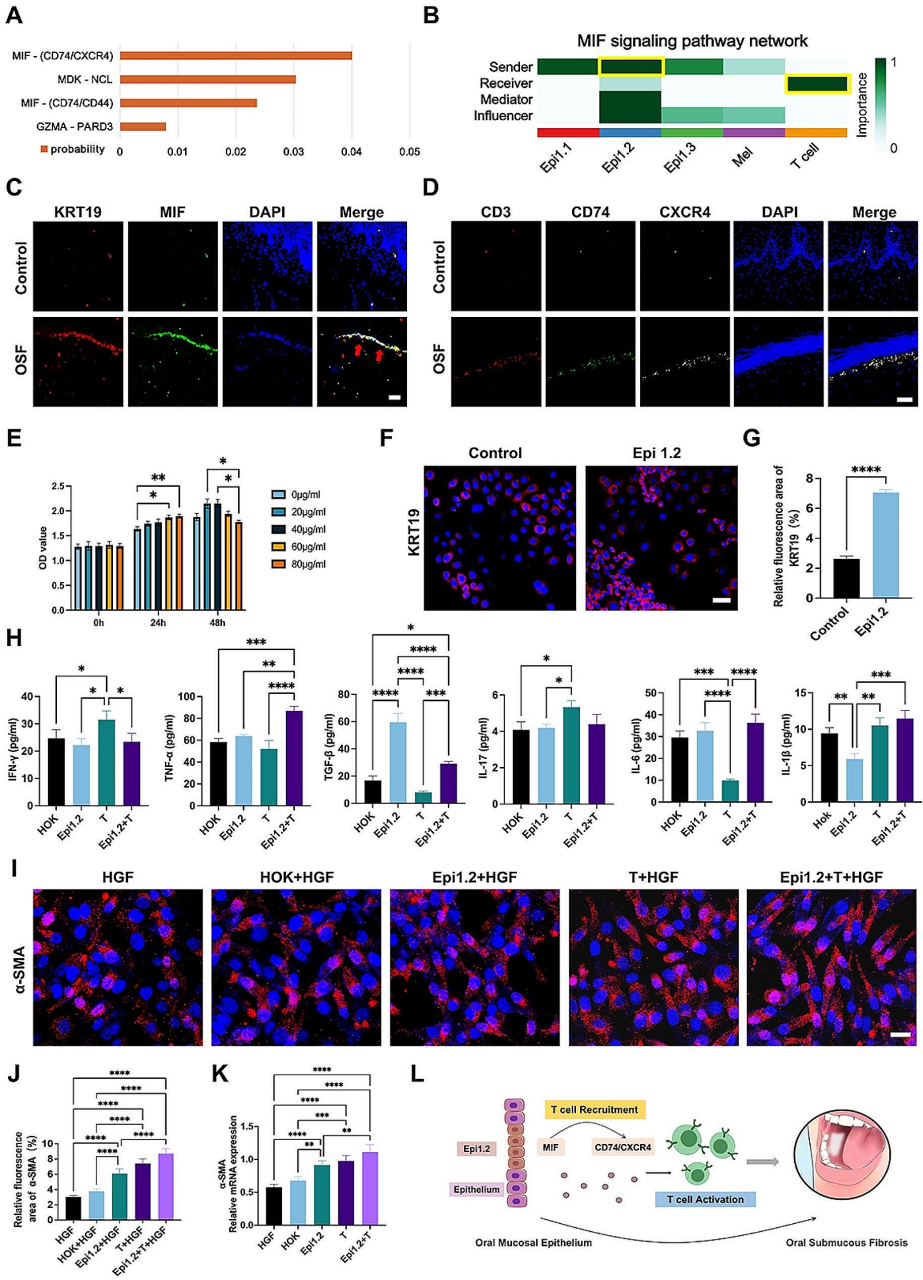
Epithelial cells interact with T cells through receptor-ligand pairs and activated fibroblasts *in vitro*. **(A)** Probability values for all receptor-ligand pairs between Epi1.2 cells and T cells. MIF, MDK, and PARD3 are ligands in Epi1.2 cells, and CD74/CXCR4, NCL, CD74/CD44, and GZMA are receptors in T cells. **(B)** The role of Epi1.2 cells and T cells in the MIF signaling pathway. Epi1.2 cells are the senders and T cells are the receivers. **(C)** Levels of Epi1.2 cells and their ligand MIF in clinical samples. The red arrows indicate the positive cells. Scale bar = 50 μm. **(D)** Levels of T cells and their receptors CD74 and CXCR4 in clinical samples. Scale bar = 20 μm. **(E)** Cell viability of epithelial cells after stimulation with arecoline (0, 20, 40, 60, and 80 µg/mL) for 0, 24, or 48 h, *n* = 3. **(F)** Immunofluorescent validation of the Epi1.2 cell model. Scale bar = 30 μm. **(G)** Quantification of the fluorescence area in the control and Epi1.2 cell model, *n* = 3. **(H)** The concentration of cytokines (IFN-gamma, TNF-α, TGF-β, IL-17, IL-6, and IL-1β) in the cell supernatant of different groups was determined using ELISA, *n* = 3. **(I)** Fluorescence images of α-SMA after treatment with different cell supernatants. HGF: human gingival fibroblasts. Scale bar = 20 μm. **(J)** Quantification of fluorescence area in **(I)**, *n* = 3. **(K)** The mRNA expression of *ACTA2* (α-SMA) after treatment with different cell supernatants, *n* = 3. **(L)** Diagrammatic representation of epithelial-T cell interactions influencing OSF progression. Epi1.2 cells interact with T cells through the ligand-receptor crosstalk of MIF-(CD74/CXCR4), activating T cells and promoting fibrosis. The results are presented as the mean ± S.D. *P < 0.05; **P < 0.01; ***P < 0.001; ****P < 0.0001

Louvain clustering analysis was conducted to identify the subpopulations of epithelial cells [27]. The analysis showed a significant diversity within the epithelial cell populations in human oral mucosal tissues, accounting for 73% of the total cell count (Appendix Fig. 2B). Further analysis of these epithelial cells identified 11 distinct cell types, termed cluster 0 to cluster 10 (Appendix Fig. 2C). Notably, in the OSF group, there was a significant increase in the number of cells belonging to subsets cluster 2, cluster 3, cluster 5, cluster 6, cluster 7, and cluster 9 (Appendix Fig. 2D). We were able to identify four subtypes based on marker gene expression, including Epi1.1, Epi1.2, Epi1.3, and a melanocyte population (Mel) (Fig. 1C). Through gene expression analysis, Epi1.1 cells include cluster 1, cluster 4, and cluster 6. Epi1.3 cells include cluster 0 and cluster 8. Interestingly, while Epi1.1 and Epi1.3 were present in both healthy and fibrotic mucosa, Epi1.2 cells (cluster 2/3/5/7/9) were mostly found in the OSF mucosa (Fig. 1C). To further confirm and visualize the presence of Epi1.2 cells, immunohistochemical staining was performed on both healthy and OSF mucosa. The marker protein KRT19, which is specific to Epi1.2 cells, was found to be expressed at significantly higher levels in the mucosa of patients with OSF compared with that in the controls (Fig. 1D).

### Epi1.2 cells, with a distinct immune and profibrotic functionality, facilitate T cell recruitment

Further clustering analysis showed that Epi1.2 cells possess a proinflammatory gene signature, including *IL6st*, *CD63*, *CD74*, *IL1r2*, *IL33*, *CCL28*, and *CXCL14*, as shown in Fig. 1E. To understand the potential developmental relationships between these subsets of epithelial cells, pseudotime analysis was performed. The results revealed that Epi1.1 and Epi1.3 cells progressed towards Epi1.2 cells in the OSF mucosa, suggesting that increased numbers of Epi1.2 cells are part of the pathogenesis of OSF (Fig. 1F; Appendix Fig. 2E). Additionally, the analysis revealed that the gene expression profile in Epi1.2 cells is consistent with inflammatory responses. This profile includes the expression of *RAI14*, *DDIT4*, *VGLL4*, *CD46*, *EIF2AK2*, and *REL* [28–32]. Furthermore, these cells were also enriched in fibrosis-associated genes, such as *TRIM2* and *REL*. This suggested that Epi1.2 cells might play a role in promoting extracellular matrix formation [32, 33] (Fig. 1F).

The results of our study and another study revealed a noticeable increase in T cells within the OSF mucosa [34] (Appendix Fig. 2F, G). Therefore, we investigated of the possible interaction between Epi1.2 and T cells in OSF, considering that Epi1.2 cells express numerous cytokines and chemokines. We further explored the cell-cell interactions within the OSF mucosa using comprehensive information on receptor-ligand binding. The cellchat analysis revealed the highest probability value for the MIF-(CD74/CXCR4) interaction, with Epi1.2 cells as the senders and T cells as the recipients of the signal (Fig. 2A, B). Specifically, MIF was found in Epi1.2 cells as a ligand, and CD74/CXCR4 was present in T cells as a receptor (Appendix Fig. 2H). The expression of MIF in Epi1.2 cells within the OSF mucosa (detected using the epithelial cell marker protein KRT19) and CD74/CXCR4, the receptors for MIF in T cells (detected using the T cell marker gene *CD3*), was confirmed through immunofluorescence in human samples (Fig. 2C, D). In summary, through subclustering, pseudotime, and cellchat analysis, as well as immunohistochemistry and immunofluorescence, we identified the presence of Epi1.2 cells with proinflammatory and profibrotic features and their interaction with T cells within the OSF mucosa. These findings suggested a unique interplay between the epithelial and stromal compartments in OSF, particularly with regard to T cells.

### Epi1.2 cells promoted an inflammatory response and fibroblast activities by interacting with T cells in the OSF cell model

To investigate the possible interaction between Epi1.2 and T cells in the development of OSF, we established an Epi1.2 cell model of OSF *in vitro* using arecoline, a primary component implicated in the progression of OSF [35, 36]. CCK8 assays and scratch tests were conducted to evaluate the impact of different concentrations of arecoline solution on the proliferation and migration of epithelial cells (HOK) over 48 h. The results showed that high concentrations of arecoline (60 µg/mL, 80 µg/mL) stimulated the proliferation of epithelial cells within the first 24 h. The proliferation ability of HOK cells could be decreased by a high concentration arecoline solution, as compared to the low concentration (20 µg/mL, 40 µg/mL) solution after 48 h (Fig. 2E). The migration ability of HOK cells could be enhanced by a high concentration arecoline solution within the first 24 h. However, they seemed to inhibit the migration after 48 h, as compared to the low concentration solution. Generally, the mobility of HOK cells was reduced when exposed to an arecoline solution (Appendix Fig. 3A, B). Based on these findings, we decided to use a concentration of 60 µg/mL and a 24 h induction period for our *in vitro* OSF cell model (Fig. 2F, G).

**Fig. 3:**
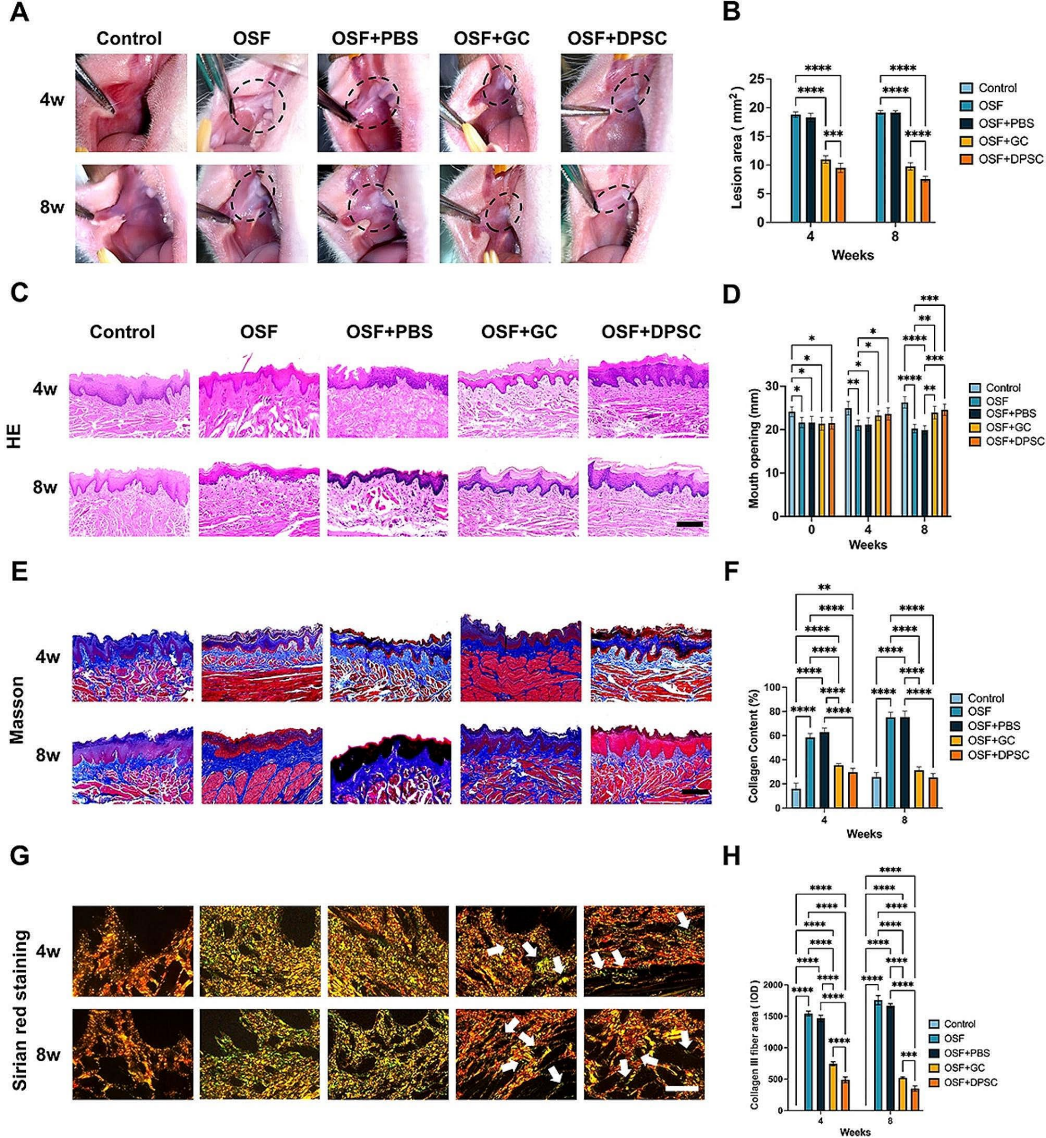
Histological analyses of the therapeutic effects of different treatments on arecoline-induced OSF rats. **(A)** Representative images of the oral mucosa of rats with different treatments (4 and 8 weeks post-injection). Control: normal rats, OSF: non-treated OSF rats, OSF + PBS: PBS injected OSF rats, OSF + GC: glucocorticoid/PBS injected OSF rats, OSF + DPSCs: dental pulp stem cells/PBS injected OSF rats. **(B)** Quantitative analysis of the lesion area (white) in **(A)**. *n* = 6. **(C)** Representative images of hematoxylin and eosin (H&E) staining for epithelial morphology at 4 and 8 weeks. Scale bar = 500 μm. **(D)** Mouth opening measurements in rats after different treatments at specified intervals (0, 4, and 8 weeks post-injection), n = 6. **(E)** Representative Masson’s trichrome images of different treatments at 4 and 8 weeks. Scale bar = 500 μm. **(F)** Quantification of the amount of collagen deposition (blue) on the submucosa in Masson’s trichrome images at 4 and 8 weeks, *n* = 6. **(G)** Representative Sirius Red images of oral mucosal observed under polarized light after different treatments at 4 and 8 weeks. The white arrows indicate positive areas. Scale bar = 50 μm. **(H)** Quantification of type III collagen (green) areas on the submucosa in Sirius Red images at 4 and 8 weeks, *n* = 6. The results are presented as the mean ± S.D. *P < 0.05; **P < 0.01; ***P < 0.001; ****P < 0.0001

To investigate further, T cells were isolated from human blood (Appendix Fig. 3C) and cocultures were conducted using the established model. Four groups were formed for this purpose: HOK, Epi1.2 cells, Epi1.2 + T cells, and T cells alone. Following incubation for 24 h, the levels of IFN-γ, TNF-α, TGF-β, IL-1β, IL-6, and IL-17 in the cell supernatant were measured using enzyme-linked immunosorbent assay (Fig. 2H). The level of TGF-β was increased, while the level of IL-1β was decreased, in Epi1.2 cells compared with those in normal epithelial cells. Additionally, T cells displayed decreased levels of IFN-γ (*p* < 0.05) and increased levels of TNF-α (*p* < 0.0001), TGF-β (*p* < 0.001), and IL-6 (*p* < 0.0001) following co-culture with Epi1.2 cells. These results indicated that the expression trend of IFN-γ, TNF-α, TGF-β, and IL-6 factors is consistent with OSF disease [4]. We then examined the expression of MIF-(CD74/CXCR4) and the activation and migration of T cells after co-culture with Epi1.2 cells. The results showed that the receptor and ligand pair existed on Epi1.2 cells and T cells, and their expression levels increased after co-culture (Appendix Fig. 3D-G). The Transwell assay showed that the migration ability of T cells was also enhanced (Appendix Fig. 3H). The expression of CD69 and CD25 on T cells rose significantly after co-culture (Appendix Fig. 3I, J). The expression of granzyme B was enhanced, but not significantly, between the two groups (Appendix Fig. 3K). The number of proliferating T cells was also augmented (Appendix Fig. 3L). To further confirm the interaction between Epi1.2 cells and T cells, the MIF inhibitor ISO-1 was used. The immunofluorescence results showed that the number of T cells and their receptors was decreased, and the migration ability of T cells was weakened by ISO-1 (Appendix Fig. 4A-C). Flow cytometry showed that the expression of CD69, CD25, and granzyme B had decreased significantly (Appendix Fig. 4D-F). The number of proliferating T cells was also decreased (Appendix Fig. 4G). These results suggested that T cells might be activated after co-culture with Epi1.2 cells.

**Fig. 4:**
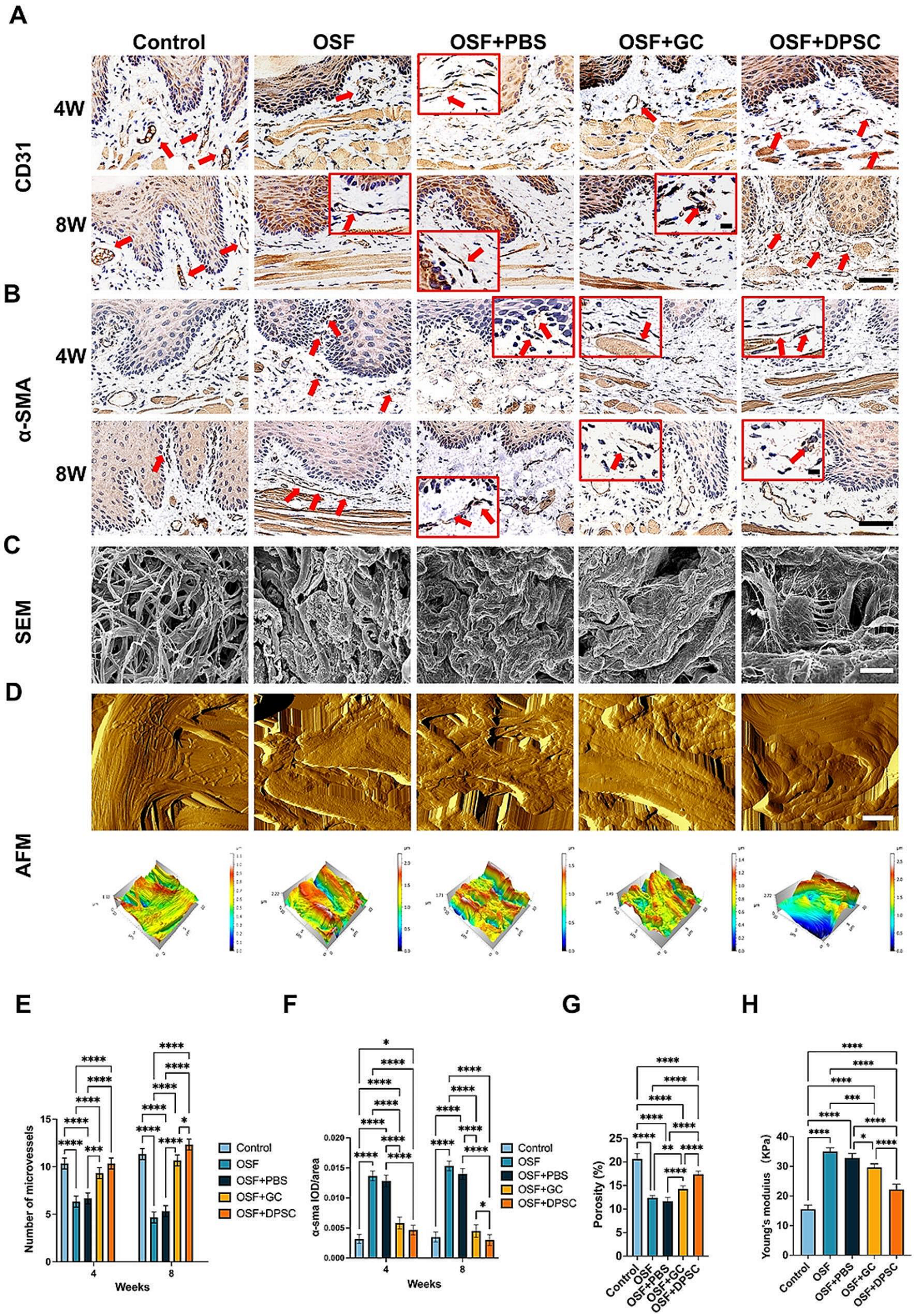
Effects of different treatments on blood vessels and myofibroblasts, and their microscopic characterization. **(A)** Immunohistochemical images of blood vessels in oral mucosa tissue at 4 and 8 weeks. Red boxes represent the locally enlarged position. Red arrows indicate blood vessels in the lamina propria of the mucosa. Scale bars = 50 μm, 20 μm. **(B)** Representative immunohistochemical images of myofibroblasts in oral mucosa tissue at 4 and 8 weeks. Red boxes represent the locally enlarged position. The red arrows indicate the positive cells. Scale bars = 50 μm, 20 μm. **(C)** Scanning electron microscopy image of the oral mucosa lamina propria of rats under different treatments (4 weeks post-injection). Scale bars = 5 μm. **(D)** Atomic force scanning topography image (above) and corresponding three-dimensional reconstruction image (below) of the oral mucosa lamina propria of rats after different treatments (4 weeks post-injection). Scale bar = 3 μm. **(E)** Quantification of the number of microvessels, *n* = 6. **(F)** Quantification of the α-SMA positive areas, *n* = 6. **(G)** Quantitative analysis of collagen porosity areas in the SEM images, *n* = 6. **(H)** Young’s modulus from the AFM images of the oral mucosa lamina propria, *n* = 6. The results are presented as the mean ± S.D. *P < 0.05; **P < 0.01; ***P < 0.001; ****P < 0.0001

Fibroblasts are key effector cells in fibrotic diseases. To further validate the scRNA-seq findings in an *in vitro* setting, HGFs were co-cultured with the supernatants of each group (HOK, Epi1.2 cells, T cells, Epi1.2 + T cells and ISO-1 + Epi1.2 + T cells) for 48 h, and the expression of α-SMA in the HGFs was detected. The results demonstrated that Epi1.2 cells, T cells, and the combination of Epi1.2 and T cells all induced upregulation of α-SMA levels (Fig. 2I-K). The ISO-1 group showed downregulation of α-SMA levels (Appendix Fig. 4H-J). These findings indicated that Epi1.2 cells might activate fibroblasts through an interaction with T cells, thereby contributing to the progression of OSF. Collectively, the epithelial cells in OSF are heterogeneous, with the presence of a pro-inflammatory and pro-fibrotic subpopulation, Epi1.2, which might communicate with T cells and promote the development of OSF (Fig. 2L).

### Oral submucous fibrosis was attenuated by local injection of DPSCs in OSF rats

Herein, the relationship between inflammation in the oral submucosa and secondary fibrosis changes was investigated using scRNA-seq and *in vitro* experiments. It was observed that DPSCs possess anti-inflammatory, anti-fibrosis, and immunomodulatory effects [37–39]. To simulate the progression from a healthy state to fibrosis, to assess the exacerbation and destructive nature of host responses and histological changes in the mucosa, and reveal the therapeutic potential of DPSCs on OSF, male Sprague-Dawley rats were used to establish an OSF animal model (Appendix Fig. 5A). This model displayed a similar white lesion area on the cheek, decreased mouth opening, and significant histological changes resembling those observed in patients with OSF (Fig. 3A-D). Post-treatment, there was a reduction in weight in the OSF, OSF + PBS, and OSF + GC groups, compared with the gradual increase observed in the OSF + DPSC group (Appendix Fig. 5B). Additionally, the OSF group showed an increased number of KRT19 ^+^ MIF ^+^ epithelial cells (Epi1.2) and receptor-ligand pairs (Appendix Fig. 5C-H).

**Fig. 5:**
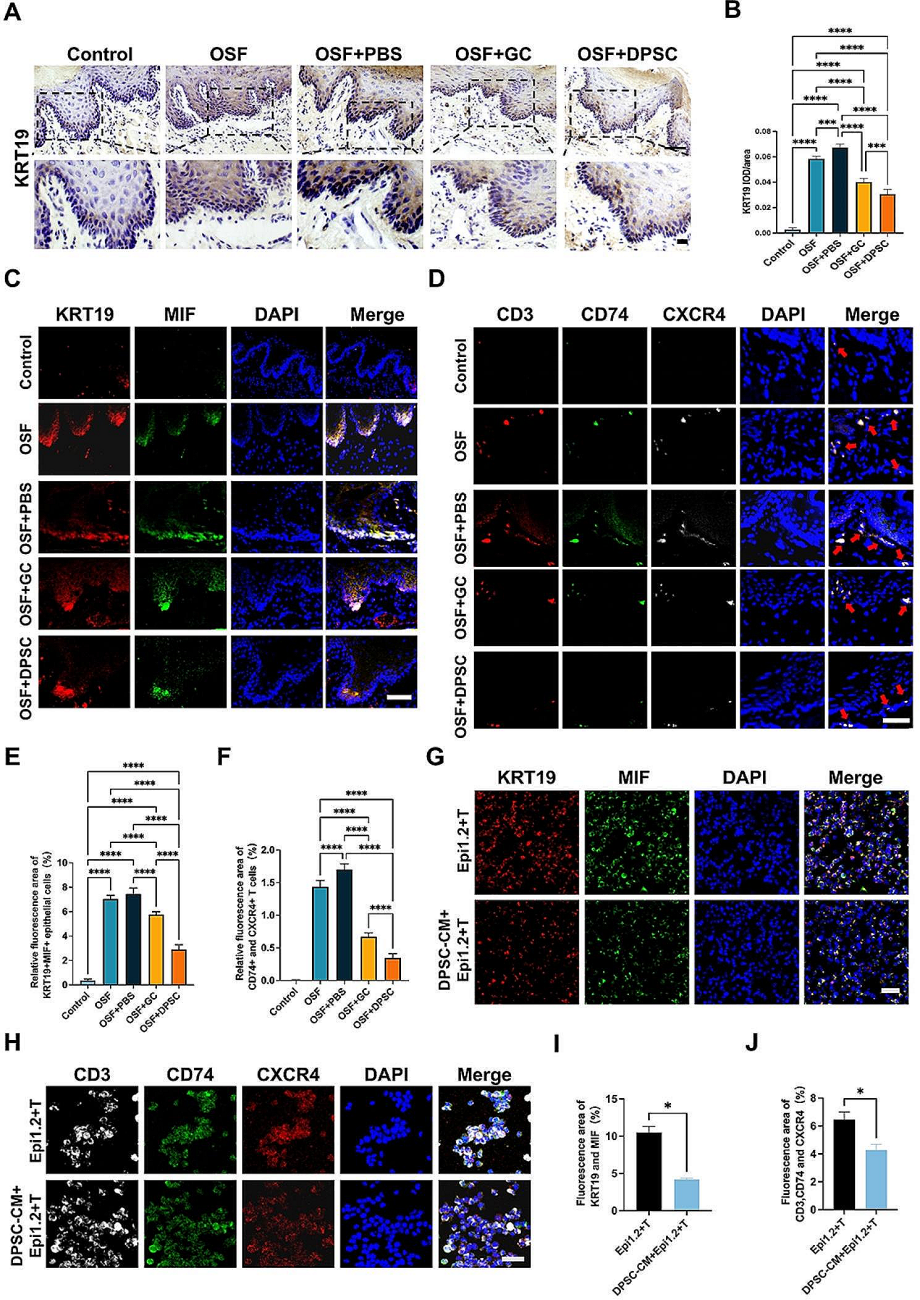
Changes in expression of KRT19 ^+^ epithelial cells and their ligand and receptor pairs after different treatments. **(A)** Representative immunohistochemical images of KRT19 ^+^ epithelial cells after different treatments (4-weeks post-injection) for OSF. The black boxes represent the locally enlarged position. Scale bars = 100 μm, 50 μm. **(B)** Quantitative analysis of the expression of KRT19 ^+^ epithelial cells in **(A)**, *n* = 6. **(C)** Representative images of KRT19 ^+^ MIF ^+^ epithelial cells after different treatments (4-weeks. post-injection) for OSF. Scale bar = 50 μm. **(D)** Representative images of immunostaining for CD74 ^+^ and CXCR4 ^+^ T cells under different treatments (4-weeks post-injection) for OSF. The red arrows indicate the positive cells. Scale bar = 50 μm. **(E&F)** Semiquantitative analysis of the percentage of the fluorescence area in Merge was performed according to the results in **(C&D)**, respectively, *n* = 6. **(G)&(H)** Representative immunofluorescence images of Epi1.2 cells cocultured with T cells and DPSC-CM *in vitr*o, *n* = 3. Scale bars = 50 μm. **(I)&(J)** Semiquantitative analysis of the fluorescence area in Merge, performed according to the results in **(G&H)**, respectively, *n* = 3. The results are presented as the mean ± S.D. *P < 0.05; ***P < 0.001; ****P < 0.0001

Following the 12-week creation period of the OSF model, the rats were divided into five groups: Control, OSF, OSF + PBS, OSF + GC, and OSF + DPSC. DPSCs and their characterization are shown in Appendix Fig. 6. Submucosal injections were administered weekly, and after 4 and 8 weeks, the animals were sacrificed for histological examination. Local submucosal GC injection is currently the primary clinical treatment for OSF. Treatment with GC and DPSCs resulted in a reduction in the area of the white lesions in the buccal area and restoration of mouth opening compared with that in the OSF group (Fig. 3A-B, D). The OSF + DPSC group showed a greater decrease in the area of white lesions compared with that in the OSF + GC group at both 4 and 8 weeks. Although there was some improvement in mouth opening, no significant difference was observed between the OSF + DPSC and OSF + GC groups. Histological examination using H&E staining demonstrated a thickened epithelial layer (Fig. 3C). Masson staining clearly showed that the overall connective tissue deposition in the oral mucosa treated with DPSCs was significantly less than that in the mucosa treated with PBS alone (Fig. 3E, F). Similarly, both the OSF + GC and OSF + DPSC groups demonstrated less deposition of type III collagen fibers (indicated by green staining), with the OSF + DPSC group showing the least deposition, as demonstrated by Sirius Red staining (Fig. 3G, H). These findings suggested that DPSCs were more effective than GC in mitigating the progression of fibrosis in rats.

**Fig. 6:**
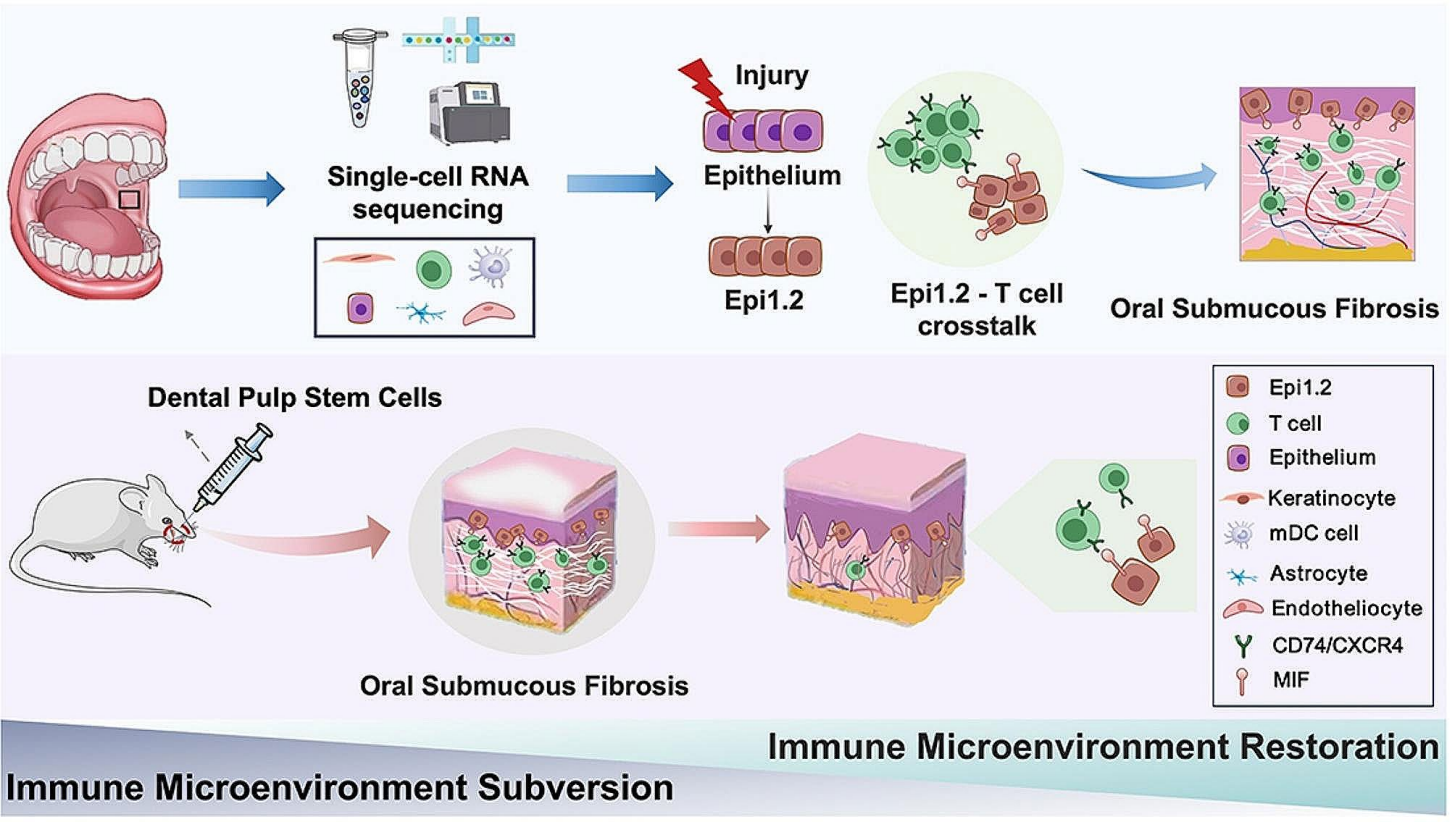
Predicted pathogenesis centered on the local microenvironment of oral submucous fibrosis and the hypothetical mechanism of DPSCs in the treatment of OSF. Single-cell RNA sequencing of buccal mucosa from healthy volunteers and OSF patients revealed a unique epithelial cell subpopulation, Epi1.2, which interacts with T cells to alter the local immune microenvironment and lead to OSF development (above). A rat model of OSF was constructed to simulate the clinical treatment. Local submucosal injection of DPSCs could slow down the progress of OSF by reducing the expression of KRT19 ^+^ MIF ^+^ epithelial cells (Epi1.2) and CD74 ^+^ and CXCR4 ^+^ T cells, restoring mucosal homeostasis, and rebuilding the local immune microenvironment (below).

Reduction in vessel number and vessel lumen diameter, and an increase in myofibroblasts, are two important pathological features of OSF. In this study, CD31 and α-SMA were used as markers to label vessels and myofibroblasts, respectively. The vessel diameter and numbers increased after treatment with GC and DPSCs (Fig. 4A, E). Furthermore, there was a reduction in myofibroblast numbers (Fig. 4B, F). Notably, the OSF + DPSC group exhibited more significant improvements after 8 weeks compared with those in the OSF + GC group.

The effectiveness of DPSC treatment was also evaluated microscopically after 4 weeks. Scanning electron microscopy analysis of tissues before and after treatment revealed an increase in porosity in both the OSF + GC and OSF + DPSC groups, indicative of a reduction in tissue density (Fig. 4C, G). Additionally, AFM was used to measure tissue hardness, which showed a decrease following treatment with OSF + GC and OSF + DPSC, with the DPSC group exhibiting more pronounced improvements (Fig. 4D, H). These results indicate that DPSCs can effectively reduce tissue density and stiffness and soften the texture of sclerotic mucosa.

Collectively, the integrated findings from morphological characterization, histological staining, immunohistochemical staining, and microscopic characterization indicated that DPSCs were more effective than GC in mitigating fibrosis progression in rats.

### DPSCs suppressed oral submucous fibrosis by regulating KRT19 ^+^ MIF ^+^ epithelial cells and their interaction with CD74 ^+^ and CXCR4 ^+^ T cells

To elucidate the mechanism by which DPSCs exert their effects, we integrated the scRNA-seq analysis results and examined the levels of KRT19 ^+^ MIF ^+^ epithelial subpopulation cells in each group of OSF rat models. After treatment with DPSCs, there was an obviously decrease in the expression of KRT19 ^+^ epithelial subpopulation cells compared with those in the OSF and OSF + GC groups (Fig. 5A, B). More KRT19 ^+^ epithelial subpopulation cells were present in OSF, which suggested that DPSCs could slow down the progression of OSF and the effect was better than that of the positive control. The immunofluorescence results confirmed a decrease in the expression of KRT19 ^+^ MIF ^+^ epithelial cells and receptors on T cells after treatment with both GC and DPSCs. There were significant differences between the two treatment groups (Fig. 5C-F). Moreover, the expression of CD74 ^+^ and CXCR4 ^+^ T cells was significantly reduced in the OSF + GC and OSF + DPSC groups compared with those in the OSF and OSF + PBS groups. Notably, the decrease was more pronounced in the OSF + DPSC group. Studies have shown that MSCs can play a therapeutic role by changing the cell phenotype, reducing the number of immune cells, or downregulating the expression of chemokines via a paracrine effect [40–42]. Therefore, after co-culture of Epi1.2 cells and T cells *in vitro*, we changed the medium to the supernatant of DPSCs to allow the three to be co-cultured. The results showed that the expression of MIF and CD74/CXCR4 on Epi1.2 cells and T cells was decreased in the group receiving DPSC-conditioned medium (DPSC-CM) group (Fig. 5G-J). These findings suggested that DPSCs might exert their therapeutic effect by decreasing the expression of KRT19 ^+^ MIF ^+^ epithelial cells and subsequently reducing the expression of recipient ligand pairs between KRT19 ^+^ MIF ^+^ epithelial cells and CD74 ^+^ and CXCR4 ^+^ T cells.

## Discussion

Epithelial cells are crucial to preserve the oral mucosa’s barrier function. Fibrosis is often seen as an outcome of unresolved inflammation, and any disruption to the epithelial barrier can trigger an inflammatory cascade [43, 44]. While fibrosis often arises from unresolved inflammation, the focus has largely been on fibroblasts, leaving the role of epithelial cells in OSF underexplored. Our study employed scRNA-seq to investigate this aspect, revealing a unique epithelial cell population, Epi1.2, and highlighting the interaction between Epi1.2 cells and T cells via MIF-(CD74/CXCR4) in OSF. Understanding the underlying mechanisms could allow intervention with a homeostatic immunoregulatory response in the context of OSF. DPSCs were shown to alleviate fibrotic symptoms, which was corroborated using an arecolineinduced rat model.

Characterizing the transcriptomic profiles of cells from normal mucosal tissues and specimens of OSF revealed significant differences in gene expression patterns [45–47]. Moreover, our scRNA-seq analysis identified a unique epithelial cell cluster in OSF, termed Epi1.2, with proinflammatory and profibrotic tendencies. Interestingly, Epi1.2 cells are found in the epithelial basement membrane, and the expression of KRT19, a marker for Epi1.2 cells, gradually increased in oral epithelial dysplasia (OED), OSCC, and malignant OSF, associated with changes in matrix stiffness [48, 49]. Therefore, it is plausible that Epi1.2 cells are involved in epithelial-mesenchymal transition (EMT) [50, 51], a hypothesis that warrants further investigation. Both scRNA-seq and histological analysis suggested an interaction between Epi1.2 cells and T cells as a potential driver of OSF. We confirmed that Epi1.2 cells and T cells can activate fibroblasts and contribute to fibrosis development *in vitro*. These findings present a new mechanism of the epithelial-immune interaction in OSF development and progression.

Mesenchymal stem cells have emerged as a promising therapeutic avenue for inflammatory and fibrotic diseases. Among them, DPSCs have also demonstrated their potential to treat various conditions [52, 53]. Herein, we investigated the use of DPSCs to treat OSF, a condition characterized by changes in the tissue immune microenvironment, through a novel target. Like DPSCs, gingival MSCs have also shown anti-inflammatory and immunomodulatory effects in treating various inflammatory and autoimmune diseases in experimental models [54–56]. Gingival tissue is relatively accessible and available following certain dental procedures, and healing typically occurs rapidly after surgery; therefore, the clinical application of GMSCs to treat inflammatory and autoimmune diseases, as well as tissue regeneration and repair, holds significant therapeutic potential. In terms of organizational sources, DPSCs are easy to isolate and expand from deciduous and permanent teeth. By contrast, obtaining healthy gum tissues can be difficult in certain situations when they are needed the most. Especially for OSF, which involves the whole oral mucosa, autologous GMSCs face a greater challenge than DPSCs in clinical application.

Based on the pathological characteristics of OSF, the inflammatory infiltration in the early stage and the deposition of collagen fibers with a decreased number of blood vessels in the late stage, the clinical strategy of combined drug delivery is usually adopted. Glucocorticoids are used to treat inflammation and reduce fibrosis, Chinese medicine such as salvia militarize and physical therapy, such as hyperbaric oxygen chambers, are used to promote blood circulation, and the whole treatment cycle should last for several months. Our study and those of other groups have shown that DPSCs have anti-inflammatory, anti-fibrotic, and pro-angiogenic effects [37–39]. Therefore, the use of DPSC treatment can not only avoid the side effects of glucocorticoids, but also reduce the concomitant medication of patients. In addition, OSF mainly affects young adults [57] with a high risk of impacted third molars. Prophylactic removal of impacted third molars can promote oral health, especially in patients with OSF with limited mouth opening [58]. At the same time, the extracted third molars can also be used as a source of autologous DPSCs to treat OSF. However, DPSCs have some shortcomings and limitations in terms of clinical feasibility, such as the therapeutic dose, the number of sources, standardized preparation, individual differences, and the underling mechanism. Although DPSCs have shown some efficacy in animal experiments, the efficacy and safety of their application in humans are still unclear. Thus, further clinical trials are needed to verify the efficacy and safety of DPSCs in humans.

While this study provided important new insights, it had some potential limitations. First, the issue of intra-patient heterogeneity could impact the generalizability of the findings. Second, the study was conducted with a limited number of samples and faced the challenge of dealing with a small number of epithelial cells and T cells in each sample. Despite these limitations, the study successfully identified the Epi1.2 cluster and demonstrated the significance of MIF-(CD74/CXCR4) in the efficacy of DPSC-mediated immune homeostasis restoration in OSF. However, further investigation is needed to understand how to alter the phenotype of Epi1.2 cells or block the binding of receptorligand pairs. Our study provides valuable insights into the mechanisms underlying OSF and highlights the translational application potential of DPSCs.

## Conclusion

In this study, we investigated the progression of OSF by analyzing normal oral mucosa and OSF specimens from individuals. By integrative scRNA-seq analysis and animal experiments, we uncovered a key mechanism that drives the development of OSF. Specifically, we found that there is abnormal crosstalk between epithelial cells and T cells, which contributes to the progression of OSF. Additionally, our research discovered that DPSCs can alleviate fibrotic diseases by inhibiting the formation of extracellular matrix components, collagen deposition, and improving local blood supply. The therapeutic effect might be exerted through paracrine effects by decreasing the expression of KRT19 ^+^ MIF ^+^ epithelial cells, which subsequently reduces the formation of recipient ligand pairs between KRT19 ^+^ MIF ^+^ epithelial cells and CD74 ^+^ and CXCR4 ^+^ T cells. This finding is significant and has profound implications for the understanding of fibrotic development. It also has the potential to inform prevention strategies and guide the development of clinical treatments for OSF.

### Electronic supplementary material

Below is the link to the electronic supplementary material.

Supplementary Material 1

Supplementary Material 2

## Data Availability

Raw and processed single-cell RNA sequencing datasets have been deposited in the NCBI GEO database under accession GSE262446.

## Abbreviations

OSF: Oral submucous fibrosis
DPSCs: Dental pulp stem cells
MIF: Macrophage migration inhibitory factor
CXCR4: C-X-C motif chemokine receptor 4
OSCC: Oral squamous cell carcinoma
GC: Glucocorticoids
MSCs: Mesenchymal stem cells
scRNA-seq: Single-cell RNA sequencing
UMI: Unique molecular identifier
t-SNE: t-distributed Stochastic Neighbor Embedding
KRT19: Keratin 19
α-SMA: Alpha smooth muscle actin
PBMCs: Peripheral blood mononuclear cells
HOK: Oral epithelial keratinocytes
HGF: Human gingival fibroblasts
H&E: Hematoxylin and eosin
Masson: Masson’s Trichrome
SEM: Scanning electron microscopy
AFM: Atomic force microscopy
GMSCs: Gingival mesenchymal stem cells
