# Supplementary material for: DPSCs regulate epithelial-T cell interactions in oral submucous fibrosis
Source: Stem Cell Res Ther. 2024 Apr 23;15:113. doi: 10.1186/s13287-024-03720-5 (PMC11036714; doi:10.1186/s13287-024-03720-5)
Supplement: Supplementary file 1 — Supplementary Material 1 [file 13287_2024_3720_MOESM1_ESM.docx]

Appendix for

**DPSCs Regulate Epithelial-T Cells Interactions in Oral Submucous Fibrosis**

S.Y. Wang ^1, #^, S.J. Zhang^2, #^, H.F. Meng^3, #^, H.Q. Xu ^1, 4^, Z.X. Guo ^1^, J.F. Yan^1^, J. L. Gao^2^, L.N. Niu ^2, *^, S.L. Wang^5,6, *^ and K. Jiao^1 *^

**Correspondence:*

1. Dr. K . Jiao,

E-mail: [kjiao1@fmmu.edu.cn](mailto:kjiao1@fmmu.edu.cn)

2. Dr. S.L. Wang,

[Email: slwang@ccmu.edu.cn](mailto:Email:%20slwang@ccmu.edu.cn))

3. Dr. L.N. Niu,

E-mail: niulina@fmmu.edu.cn

**This file includes:**

Supplementary Table 1

Supplementary Figures and Legends

**Supplementary Table 1**

**Characteristics of human samples**

| **Sample ID** | **Tissue location** | **Disease status** | **Assay** | **Sex** | **Race** | **Age** |
| --- | --- | --- | --- | --- | --- | --- |
| SH99449 | buccal | health | scRNA Seq | F | Asian | 23 |
| SO44884 | buccal | OSF | scRNA Seq | M | Asian | 24 |
| HO23838 | buccal | OSF | Histological staining | M | Asian | 29 |
| HH39760 | buccal | health | Histological staining | F | Asian | 58 |
| HO43888 | buccal | OSF | Histological staining | M | Asian | 26 |
| HO44469 | buccal | OSF | Histological staining | M | Asian | 31 |
| HO11055 | buccal | OSF | Histological staining | M | Asian | 26 |
| HO53469 | buccal | OSF | Histological staining | M | Asian | 23 |
| HO78321 | buccal | OSF | Histological staining | M | Asian | 58 |
| HO96562 | buccal | OSF | Histological staining | M | Asian | 41 |
| HO80279 | buccal | OSF | Histological staining | F | Asian | 55 |
| HO96585 | buccal | OSF | Histological staining | M | Asian | 40 |
| HO44592 | buccal | OSF | Histological staining | M | Asian | 49 |
| HO11193 | buccal | OSF | Histological staining | M | Asian | 22 |
| HO53098 | buccal | OSF | Histological staining | F | Asian | 28 |
| HO09951 | buccal | OSF | Histological staining | M | Asian | 25 |
| HO43298 | buccal | OSF | Histological staining | M | Asian | 32 |
| HO54416 | buccal | OSF | Histological staining | M | Asian | 33 |
| HO54793 | buccal | OSF | Histological staining | M | Asian | 29 |
| HO59569 | buccal | OSF | Histological staining | M | Asian | 26 |
| HO60145 | buccal | OSF | Histological staining | M | Asian | 32 |
| HH77294 | buccal | health | Histological staining | F | Asian | 29 |
| HH86329 | buccal | health | Histological staining | F | Asian | 24 |
| HH95524 | buccal | health | Histological staining | M | Asian | 80 |
| HH77272 | buccal | health | Histological staining | M | Asian | 49 |
| BF10872 | blood | health | Flow cytometer | F | Asian | 25 |
| BF32479 | blood | health | Flow cytometer | F | Asian | 34 |
| BF95103 | blood | health | Flow cytometer | M | Asian | 33 |
| BF64298 | blood | health | Flow cytometer | M | Asian | 26 |

**Supplementary Figures and Legends**

**
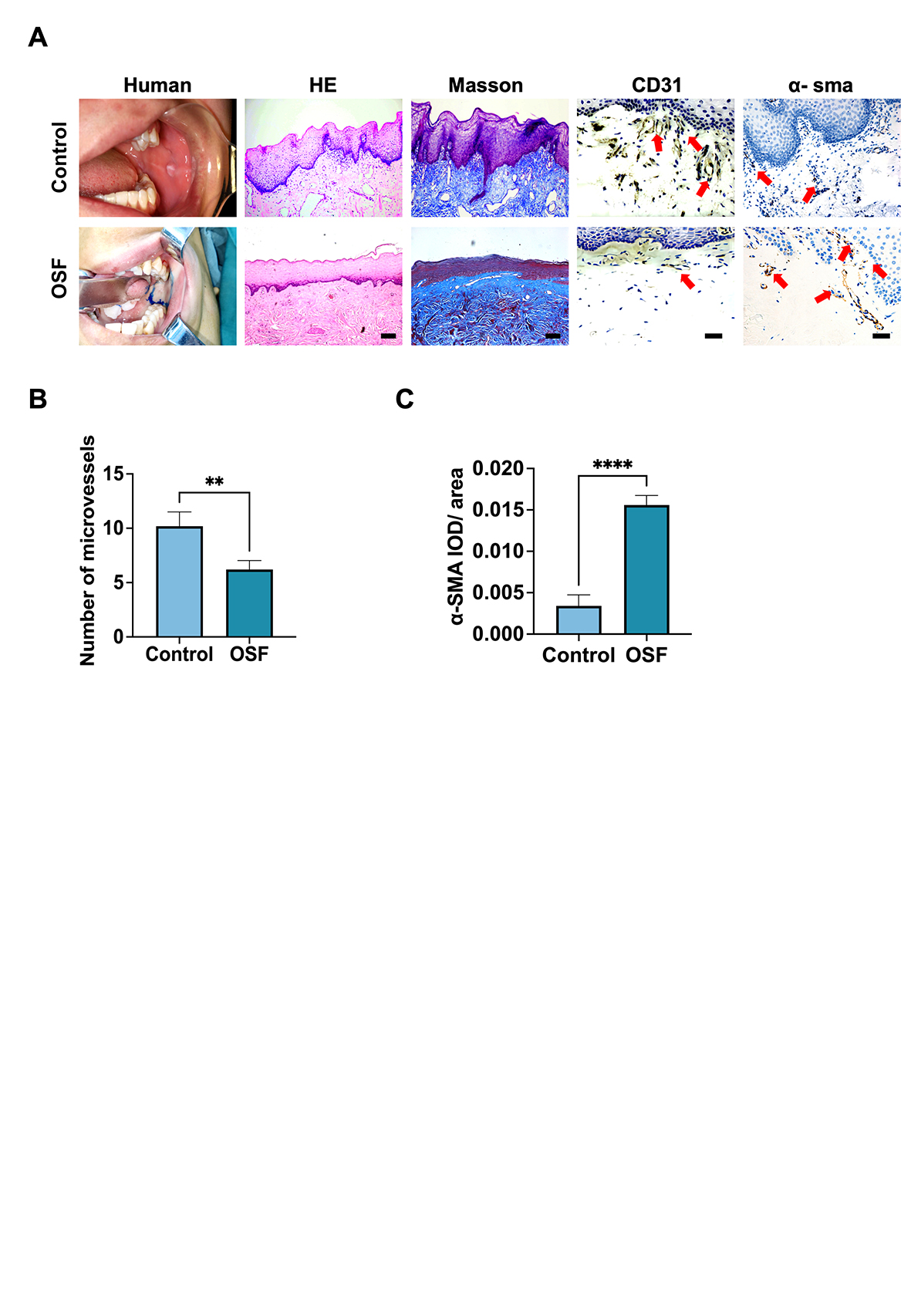
**

**Supplemental Figure 1. Histological characterization of OSF.**

**(A).** Histological characterization of clinical samples for scRNA-seq. The data include samples of buccal mucosa for lesion presentation, H&E staining for epithelial morphology, and Masson staining for collagen deposition. Scale bars = 200 μm. Immunohistochemistry for vascular and myofibroblast profiles. The red arrows indicate the positive cells. Scale bars = 50 μm, n = 6. **(B).** Quantification of the number of microvessels in **(A)**, n = 6. **(C).** Quantification of the positive areas of a-SMA in **(A)**, n = 6. Results are presented as the mean ± S.D. ***P* < 0.01; *****P* < 0.0001.

**
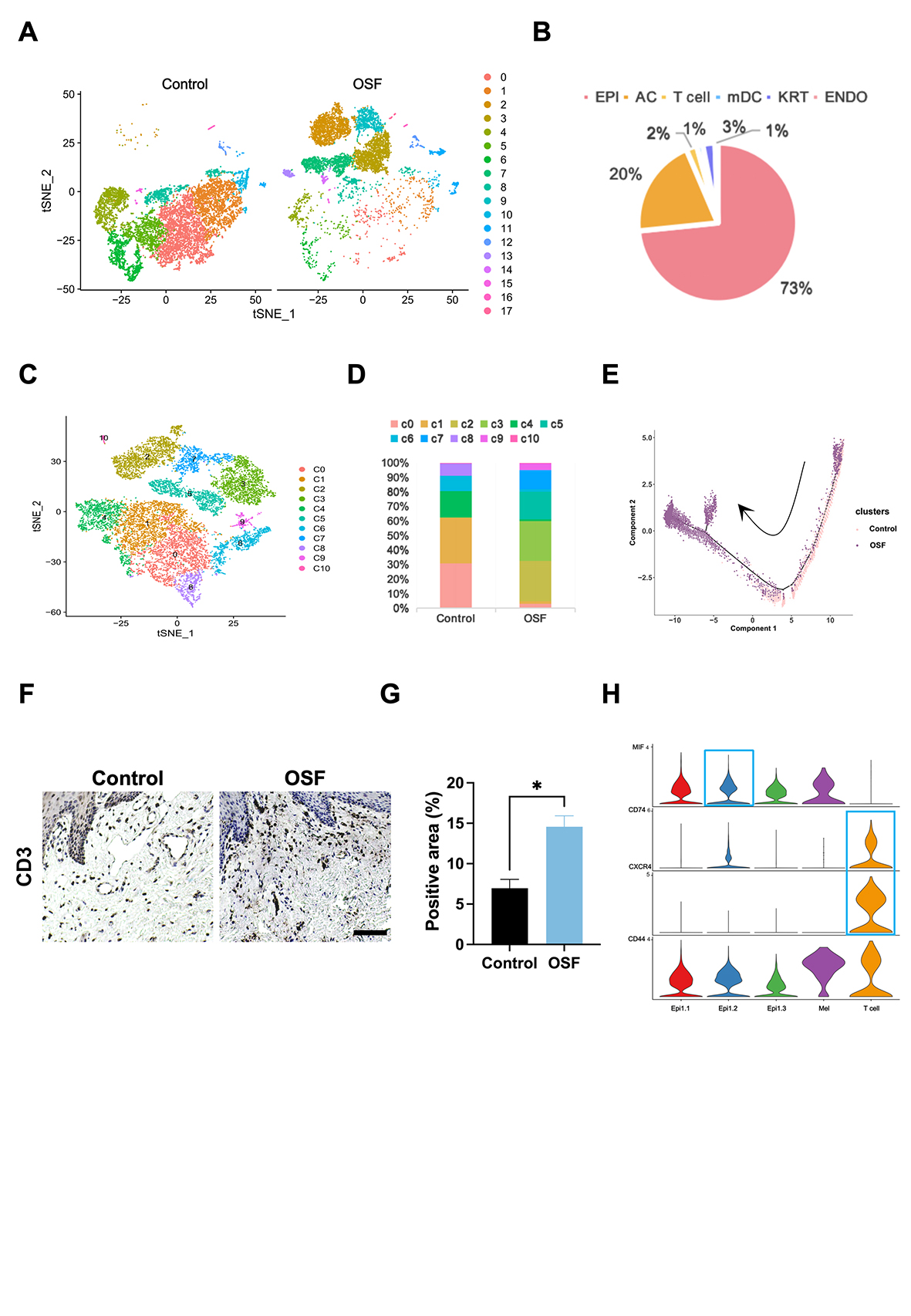
**

**Supplemental Figure 2. Overview of single-cell RNA sequencing and the expression of T cells in the submucosa.**

**(A).** The tSNE plots illustrating the representation of 18 identified clusters of cell types from oral mucosa samples of one donor/group in clinical studies. **(B)**. Bar graph of the proportions by cell type. Upper: The definition of the identified cell types, including epithelial cells, astrocytes, T cells, myeloid dendritic cells (mDCs), keratinocytes, and endothelial cells. **(C)**. The tSNE plots of epithelial cells (a total of 11 clusters). **(D)**. The proportion of epithelial cell subclusters in the two samples. **(E)**. Pseudotime trajectory plots for the epithelial cells of the two samples. The black arrow points to the differentiation trajectories of epithelial cells. **(F).** Immunohistochemical staining of clinicopathological sections of T cells. Scale bar = 100 μm. **(G).** Quantification of the positive areas in **(F)**, n = 6. **(H).** Violin plot of Epi1.2 cells and T cells receptor‑ligand pair distribution. MIF is a ligand located on Epi1.2 cells, and CD74/CXCR4 are receptors located on T cells. The blue boxes show the ligand and receptors. The results are presented as the mean ± S.D. **P* < 0.05.


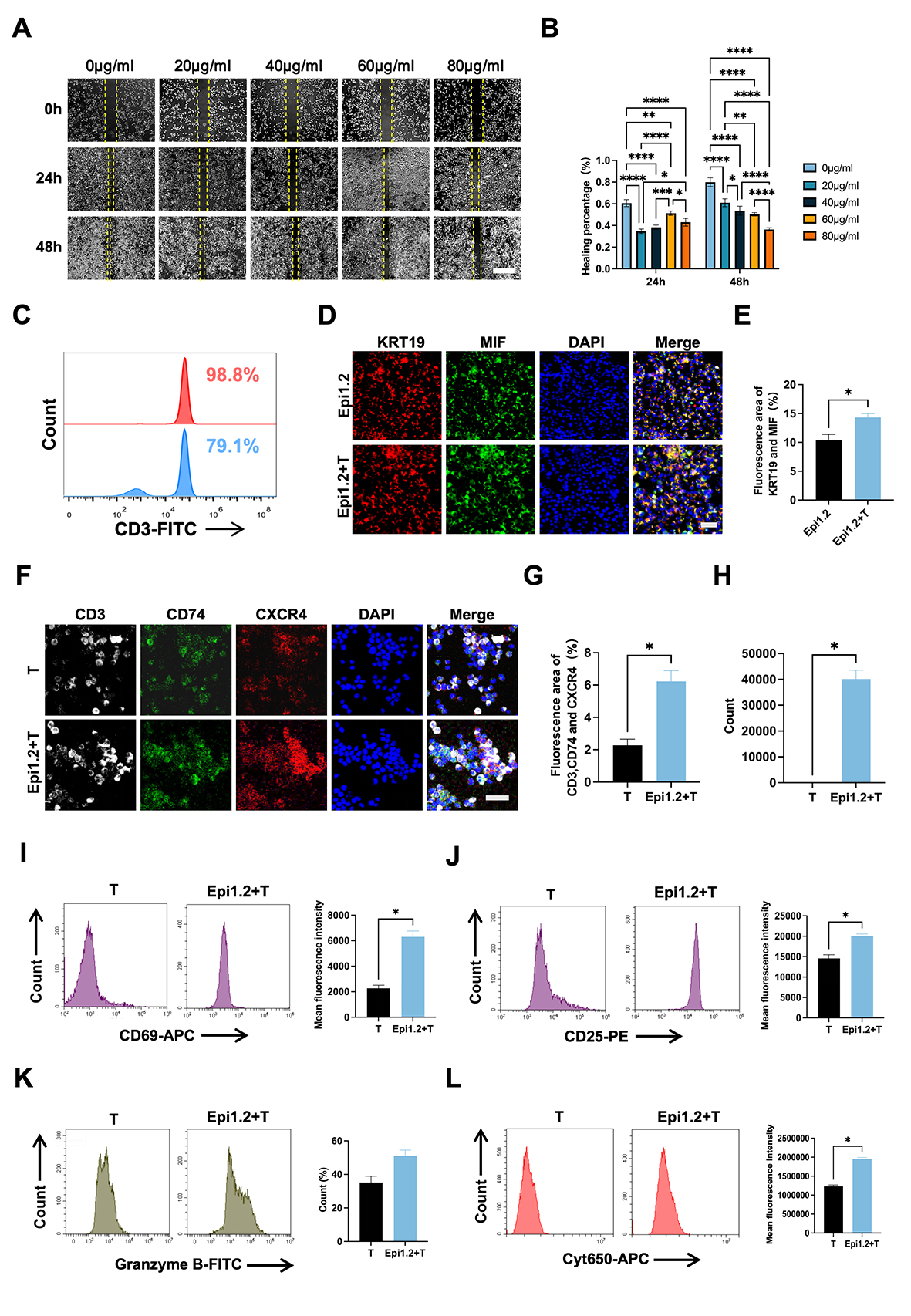


**Supplemental Figure 3.** **Effect of arecoline on epithelial cell migration and the activation of T cells after co-culture with Epi1.2 cells.**

**(A)**. Cell migration of epithelial cells after stimulation with arecoline (0, 20, 40, 60, and 80 μg/mL) for 24 and 48 h. The distance between the two yellow lines indicates the width of the scratch. Scale bar = 500 μm. **(B)**. Semiquantitative analysis according to the results of cell migration, n = 3. **(C)**. The flow cytometry results of CD3+T cells isolated from PBMCs. The percentage of CD3+T cells in PBMCs was 79.1%. CD3+ T cells accounted for 98.8% after sorting. **(D)**. Immunofluorescence images of Epi1.2 cells before and after co-culture with T cells. KRT19 is a marker of Epi1.2 and MIF is a ligand of Epi1.2 cells. Scale bar = 50 μm. **(E)** Quantification of the fluorescence area in **(D)**, n = 3. **(F)** Immunofluorescence images of T cells before and after co-culture with Epi1.2 cells. CD3 is a marker of T cells and CD74/CXCR4 is the receptor of T cells. Scale bar = 50 μm. **(G)** Quantification of the fluorescence area in **(F)**, n = 3. **(H)** Quantitative analysis of T cell migration, n = 3. **(I-L)** Flow cytometry and quantitative analysis of CD69, CD25, and granzyme B, and the proliferation of T cells, n = 3. The results are presented as the mean ± S.D. **P* < 0.05; ***P* < 0.01; *****P* < 0.0001.


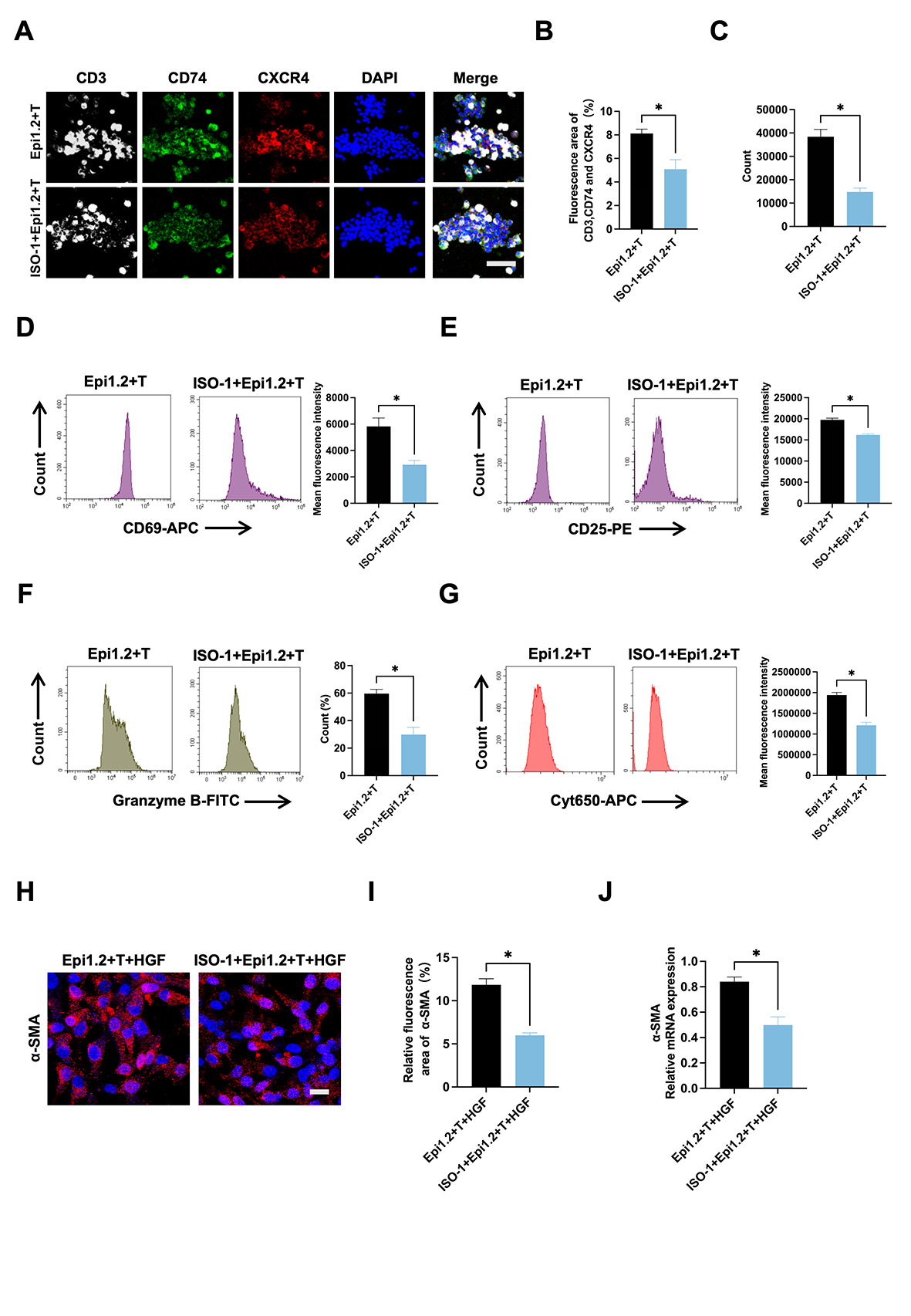


**Supplemental Figure 4. *In vitro* inhibition assay of Epi1.2 cells.**

1. . T cells co‑cultured with Epi1.2 cells after the addition of MIF inhibitor ISO-1. Scale bar = 50 μm. **(B)** Quantification of the fluorescence area in **(A)**, n = 3. **(C)** Quantitative analysis of T cell migration, n = 3. **(D-G)** Flow cytometry and quantitative analysis of CD69, CD25, and granzyme B, and the proliferation of T cells after the addition of ISO-1. n = 3. **(H)** Fluorescence images of α-SMA after treatment with different cell supernatants. HGF: human gingival fibroblasts. Scale bar = 50 μm. **(I)** Quantification of the fluorescence area in **(H)**, n = 3. **(J)** The mRNA expression of *ACTA2* (α-SMA) after treatment with different cell supernatants, n = 3. The results are presented as the mean ± S.D. **P* < 0.05.


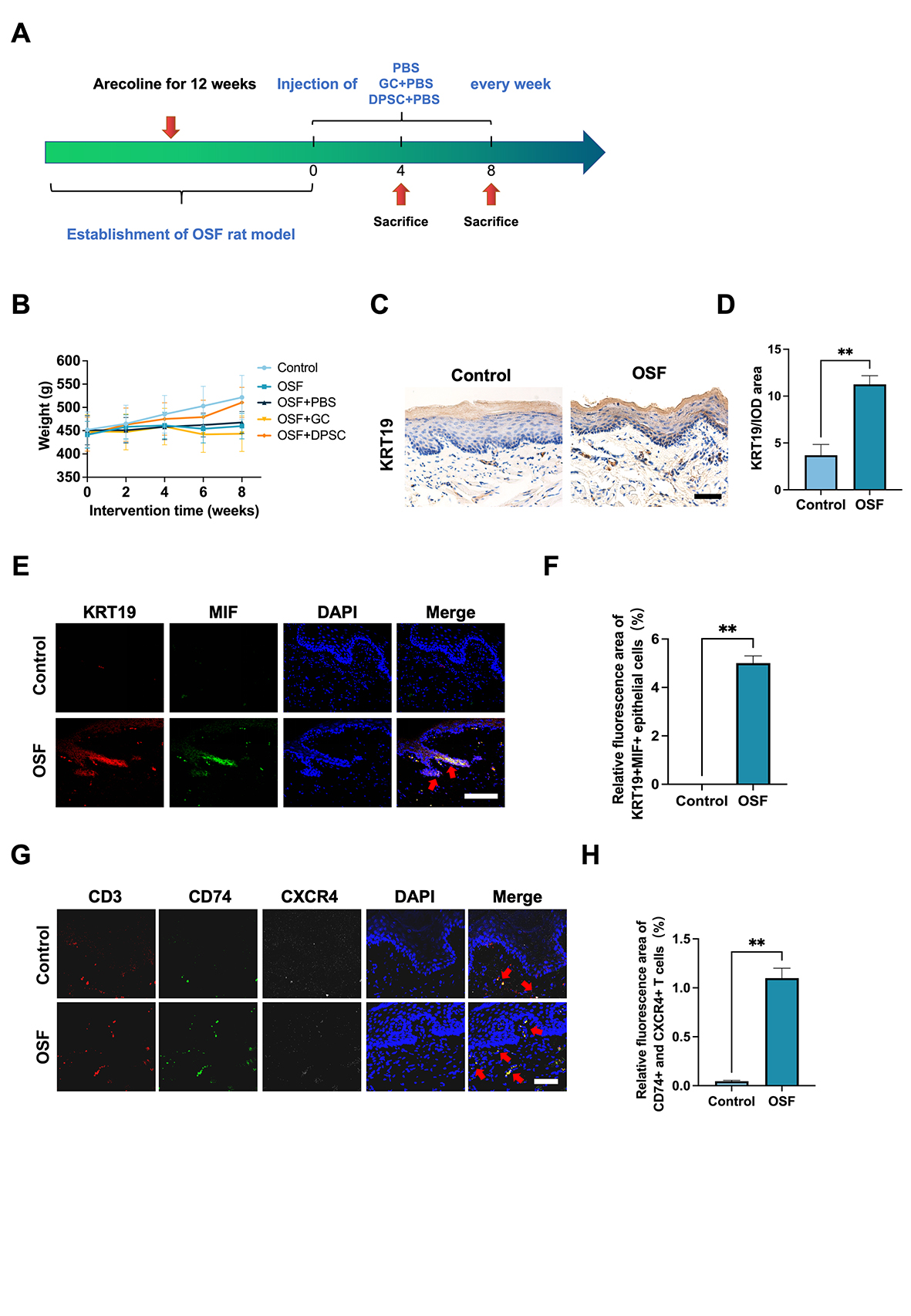


**Supplemental Figure 5. The validation of scRNA-seq results in the OSF rat models.**

**(A)**. Schematic diagram of OSF rat models establishment and treatment. Eight-week-old male Sprague-Dawley rats were used in the experiments and examined at eight to twelve weeks of age until a stable white lesion appeared. The OSF animal models were then randomly divided into four intervention groups: OSF, OSF+PBS, OSF+GC/PBS, and OSF+DPSC/PBS (n = 6). The drug was injected once a week, and the animals were sacrificed at 4 and 8 weeks after the injection. **(B)**. The weight change trend in rats with different treatments from 0 to 8 weeks post-injection. n = 6. **(C)**. Expression of KRT19+ epithelial cells in OSF rat models. Scale bar = 50 μm. **(D)**. Quantitative analysis of the positive area shown in **(C)**, n = 6. **(E)**. Expression of KRT19+MIF+ epithelial cells in OSF rat models. The red arrows indicate the positive cells. Scale bar = 50 μm. **(F)**. Quantification of the fluorescence area in **(E)**, n = 6. **(G)**. Expression of CD74+ and CXCR4+ T cells in the OSF rat models. The red arrows indicate the positive cells. Scale bar = 50 μm. **(H)**. Quantification of the fluorescence area in **(G)**, n = 6. The results are presented as the mean ± S.D. ***P* < 0.01.


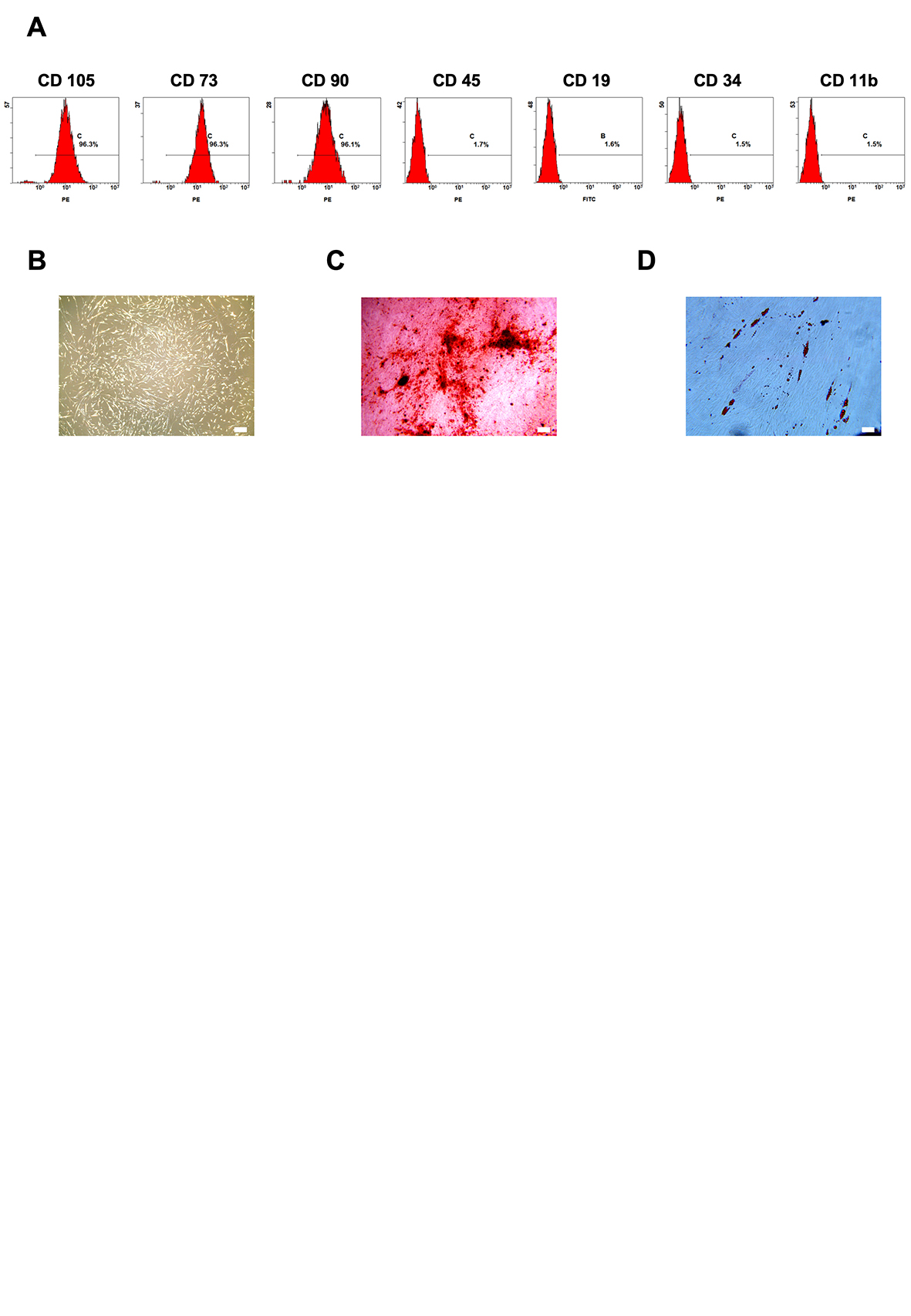


**Supplemental Figure 6. Identification of DPSCs.**

**(A)**. Results of flow cytometry phenotype identification of DPSCs. **(B)**. Morphology of DPSCs under light microscopy. Scale bar = 200 μm. **(C)**. Images of DPSCs induced for osteogenic differentiation. Scale bar = 200 μm. **(D)**. Image of DPSCs induced for adipogenic differentiation. Scale bar = 200 μm.
