# Supplementary material for: DPSCs regulate epithelial-T cell interactions in oral submucous fibrosis
Source: Stem Cell Res Ther. 2024 Apr 23;15:113. doi: 10.1186/s13287-024-03720-5 (PMC11036714; doi:10.1186/s13287-024-03720-5)
Supplement: Supplementary file 2 — Supplementary Material 2 [file 13287_2024_3720_MOESM2_ESM.doc]

STROBE Statement—checklist of items that should be included in reports of observational studies

|  | Item No | Recommendation |
| --- | --- | --- |
| **Title and abstract**  **Page 3, lines 56-59 and 62-65** | 1 | 1. Indicate the study’s design with a commonly used term in the title or the abstract |
|  |  | 1. Provide in the abstract an informative and balanced summary of what was done and what was found |
| Introduction | | |
| Background/rationale  **Pages 4-5, lines 86-90** | 2 | Explain the scientific background and rationale for the investigation being reported |
| Objectives  **Page 5, lines 103-106** | 3 | State specific objectives, including any prespecified hypotheses |
| Methods | | |
| Study design  **Pages 6-7, lines 115-132**  **Page 2, table1 in appendix** | 4 | Present key elements of study design early in the paper |
| Setting  **Pages 6-7, lines 115-132**  **No need for follow-up** | 5 | Describe the setting, locations, and relevant dates, including periods of recruitment, exposure, follow-up, and data collection |
| Participants  **Pages 6-7, lines 115-132** | 6 | (*a*) *Cohort study*—Give the eligibility criteria, and the sources and methods of selection of participants. Describe methods of follow-up  *Case-control study*—Give the eligibility criteria, and the sources and methods of case ascertainment and control selection. Give the rationale for the choice of cases and controls  *Cross-sectional study*—Give the eligibility criteria, and the sources and methods of selection of participants |
|  |  | (*b*) *Cohort study*—For matched studies, give matching criteria and number of exposed and unexposed  *Case-control study*—For matched studies, give matching criteria and the number of controls per case |
| Variables  **Pages 6-7, lines 115-124** | 7 | Clearly define all outcomes, exposures, predictors, potential confounders, and effect modifiers. Give diagnostic criteria, if applicable |
| Data sources/ measurement  **Page 6, lines 121-128**  **Pages 7-12, lines 134-248** | 8* | For each variable of interest, give sources of data and details of methods of assessment (measurement). Describe comparability of assessment methods if there is more than one group |
| Bias  **Pages 8-9, lines 171-189** | 9 | Describe any efforts to address potential sources of bias |
| Study size  **Page 6, lines 115-122** | 10 | Explain how the study size was arrived at |
| Quantitative variables  **According to the diagnostic criteria, the patients were divided into OSF group and healthy group** | 11 | Explain how quantitative variables were handled in the analyses. If applicable, describe which groupings were chosen and why |
| Statistical methods  **Page 20, lines 424-429** | 12 | (*a*) Describe all statistical methods, including those used to control for confounding |
|  |  | (*b*) Describe any methods used to examine subgroups and interactions |
|  |  | (*c*) Explain how missing data were addressed |
|  |  | (*d*) *Cohort study*—If applicable, explain how loss to follow-up was addressed  *Case-control study*—If applicable, explain how matching of cases and controls was addressed  *Cross-sectional study*—If applicable, describe analytical methods taking account of sampling strategy |
|  |  | (*e*) Describe any sensitivity analyses |

Continued on next page

| Results | | |
| --- | --- | --- |
| Participants  **Page 6, lines 115-124** | 13* | (a) Report numbers of individuals at each stage of study—eg numbers potentially eligible, examined for eligibility, confirmed eligible, included in the study, completing follow-up, and analysed |
|  |  | (b) Give reasons for non-participation at each stage |
|  |  | (c) Consider use of a flow diagram |
| Descriptive data  **Page 2, table1 in appendix** | 14* | (a) Give characteristics of study participants (eg demographic, clinical, social) and information on exposures and potential confounders |
|  |  | (b) Indicate number of participants with missing data for each variable of interest |
|  |  | (c) *Cohort study*—Summarise follow-up time (eg, average and total amount) |
| Outcome data  **Page 2, table1 in appendix**  **Figure legends in**  **Figure 1 and supplemental Figure 1-4 in appendix** | 15* | *Cohort study*—Report numbers of outcome events or summary measures over time |
|  |  | *Case-control study—*Report numbers in each exposure category, or summary measures of exposure |
|  |  | *Cross-sectional study—*Report numbers of outcome events or summary measures |
| Main results  **Figure 2 G, H, J and K;**  **Figure 1-4 in appendix** | 16 | (*a*) Give unadjusted estimates and, if applicable, confounder-adjusted estimates and their precision (eg, 95% confidence interval). Make clear which confounders were adjusted for and why they were included |
|  |  | (*b*) Report category boundaries when continuous variables were categorized |
|  |  | (*c*) If relevant, consider translating estimates of relative risk into absolute risk for a meaningful time period |
| Other analyses  **Pages 20-22, lines 432-465** | 17 | Report other analyses done—eg analyses of subgroups and interactions, and sensitivity analyses |
| Discussion | | |
| Key results  **Page 29,**  **lines 618-623** | 18 | Summarise key results with reference to study objectives |
| Limitations  **Page 31, lines 666-673** | 19 | Discuss limitations of the study, taking into account sources of potential bias or imprecision. Discuss both direction and magnitude of any potential bias |
| Interpretation  **Pages 29-30, lines 624-635** | 20 | Give a cautious overall interpretation of results considering objectives, limitations, multiplicity of analyses, results from similar studies, and other relevant evidence |
| Generalisability  **Page 30,**  **lines 633-635** | 21 | Discuss the generalisability (external validity) of the study results |
| Other information | | |
| Funding  **Page 34,**  **lines 718-722** | 22 | Give the source of funding and the role of the funders for the present study and, if applicable, for the original study on which the present article is based |

*Give information separately for cases and controls in case-control studies and, if applicable, for exposed and unexposed groups in cohort and cross-sectional studies.

**Note:** An Explanation and Elaboration article discusses each checklist item and gives methodological background and published examples of transparent reporting. The STROBE checklist is best used in conjunction with this article (freely available on the Web sites of PLoS Medicine at http://www.plosmedicine.org/, Annals of Internal Medicine at http://www.annals.org/, and Epidemiology at http://www.epidem.com/). Information on the STROBE Initiative is available at www.strobe-statement.org.
